# MerTK expressing hepatic macrophages promote the resolution of inflammation in acute liver failure

**DOI:** 10.1136/gutjnl-2016-313615

**Published:** 2017-04-27

**Authors:** Evangelos Triantafyllou, Oltin T Pop, Lucia A Possamai, Annika Wilhelm, Evaggelia Liaskou, Arjuna Singanayagam, Christine Bernsmeier, Wafa Khamri, Gemma Petts, Rebecca Dargue, Scott P Davies, Joseph Tickle, Muhammed Yuksel, Vishal C Patel, Robin D Abeles, Zania Stamataki, Stuart M Curbishley, Yun Ma, Ian D Wilson, Muireann Coen, Kevin J Woollard, Alberto Quaglia, Julia Wendon, Mark R Thursz, David H Adams, Chris J Weston, Charalambos G Antoniades

**Affiliations:** 1 Institute of Liver Studies, King's College Hospital, King's College London, London, UK; 2 Division of Digestive Diseases, St Mary's Hospital, Imperial College London, London, UK; 3 National Institute for Health Research Birmingham Liver Biomedical Research Unit, Institute of Immunology and Immunotherapy, University of Birmingham, Birmingham, UK; 4 Division of Computational and Systems Medicine, Department of Surgery and Cancer, Imperial College London, London, UK; 5 Division of Immunology and Inflammation, Department of Medicine, Imperial College London, London, UK

**Keywords:** MACROPHAGES, ACUTE LIVER FAILURE, INFLAMMATION, IMMUNOLOGY

## Abstract

**Objective:**

Acute liver failure (ALF) is characterised by overwhelming hepatocyte death and liver inflammation with massive infiltration of myeloid cells in necrotic areas. The mechanisms underlying resolution of acute hepatic inflammation are largely unknown. Here, we aimed to investigate the impact of Mer tyrosine kinase (MerTK) during ALF and also examine how the microenvironmental mediator, secretory leucocyte protease inhibitor (SLPI), governs this response.

**Design:**

Flow cytometry, immunohistochemistry, confocal imaging and gene expression analyses determined the phenotype, functional/transcriptomic profile and tissue topography of MerTK+ monocytes/macrophages in ALF, healthy and disease controls. The temporal evolution of macrophage MerTK expression and its impact on resolution was examined in APAP-induced acute liver injury using wild-type (WT) and Mer-deficient (Mer^−/−^) mice. SLPI effects on hepatic myeloid cells were determined in vitro and in vivo using APAP-treated WT mice.

**Results:**

We demonstrate a significant expansion of resolution-like MerTK+HLA-DR^high^ cells in circulatory and tissue compartments of patients with ALF. Compared with WT mice which show an increase of MerTK+MHCII^high^ macrophages during the resolution phase in ALF, APAP-treated Mer^−/−^ mice exhibit persistent liver injury and inflammation, characterised by a decreased proportion of resident Kupffer cells and increased number of neutrophils. Both in vitro and in APAP-treated mice, SLPI reprogrammes myeloid cells towards resolution responses through induction of a MerTK+HLA-DR^high^ phenotype which promotes neutrophil apoptosis and their subsequent clearance.

**Conclusions:**

We identify a hepatoprotective, MerTK+, macrophage phenotype that evolves during the resolution phase following ALF and represents a novel immunotherapeutic target to promote resolution responses following acute liver injury.

**Significance of this study**

**What is already known on this subject?**

- Liver inflammation is central to the pathogenesis of acute liver failure (ALF) where infiltration of myeloid cells in areas of hepatic necrosis is contrasted by systemic immune cell depletion and dysregulation.
- Mer tyrosine kinase ( MerTK) regulates innate immune responses and promotes the clearance of apoptotic cells following acute tissue injury.
- Secretory leucocyte protease inhibitor (SLPI) is produced within the inflamed liver in ALF and is a key modulator of monocyte anti-inflammatory responses.

**What are the new findings?**

- Patients with ALF have an expansion of resolution-like MerTK+HLA-DR^high^ monocytes and hepatic macrophages, characterised by suppressed innate and enhanced efferocytic/phagocytic responses.
- MerTK+ monocytes exhibit a distinct pattern of adhesion, phagocytosis, pattern-recognition, and cytokine receptors and genes associated with antigen presentation and macrophage polarisation.
- A similar phenotype (MerTK+MHCII^high^) with enhanced phagocytic capabilites evolves during the resolution phase of APAP-induced acute liver injury in mice.
- MerTK-deficient mice exhibit persistent liver injury and inflammation after APAP overdose and are characterised by a depletion in MHCII^high^-bearing prorestorative resident Kupffer cells and by increased numbers of activated neutrophils.
- SLPI reprogrammes myeloid cells towards resolution responses by inducing the prorestorative MerTK+HLA-DR^high^ phenotype which promotes neutrophil apoptosis and their subsequent clearance.

**How might it impact on clinical practice in the foreseeable future?**

- SLPI is a pivotal proresolving mediator in ALF that promotes MerTK-dependent hepatic resolution responses following acute liver injury.
- Harnessing the prorestorative capabilities of MerTK+ cells represents a novel therapeutic strategy to promote resolution following acute hepatic inflammmtory disorders.

## Introduction

Acute liver failure (ALF) is a clinical syndrome caused by overwhelming hepatocyte death, often leading to jaundice, encephalopathy and multiorgan dysfunction.^1^ Drug-induced liver injury, particularly acetaminophen (APAP)-induced ALF (AALF), is the most common cause of ALF, and despite liver transplantation as an option it has a high mortality rate.^1^ Although the inciting event in ALF is hepatocellular death, mortality is a consequence of activation of systemic inflammatory responses (SIRS) and its attendant complications of multi-organ failure and recurrent infection, generated by uncontrolled immune-mediated liver injury.^2^ ^3^

Central to the pathogenesis of ALF is liver inflammation where the infiltration of myeloid cells in areas of centrilobular necrosis is contrasted by immune cell depletion and dysregulation.^4^ Due to their inherent plasticity, monocytes/macrophages execute diverse functions during tissue inflammation, at both initiation and resolution phases, and exist in numerous activation states influenced by different microenvironmental cues.^5^ At steady state, monocytes traffic to the liver, augmenting the local macrophage pool, a process that is markedly increased during ALF.^6^ ^7^ Patients with ALF exhibit an expansion of hepatic macrophages, localised in areas of necrosis, through chemokine-dependent recruitment of monocyte-derived macrophages (MoMF) and proliferation of resident Kupffer cells (KCs).^4^ ^6–10^ Studies in humans and mice indicate that hepatic macrophages orchestrate both tissue-destructive and resolution/repair responses following acute liver injury.^4^ ^6^ ^10^ ^11^ Both resident KCs and MoMF populations are important effectors of resolution/tissue-repair processes following acute liver injury, where, in the absence of both or either of these phagocytes, there is significantly impaired recovery and clearance of intrahepatic neutrophils.^6^ ^7^ ^12^ ^13^ ^14^

Mer tyrosine kinase (MerTK) is a member of the Tyro3-Axl-MerTK (TAM) family of receptor tyrosine kinases expressed predominantly on macrophages.^15^ Following acute tissue injury, MerTK dampens innate immune responses and promotes clearance (efferocytosis) of apoptotic cells.^15^ Engagement and activation of MerTK inhibit signalling pathways triggered by cytokines and toll-like receptor ligands through suppressor of cytokine signaling protein (SOCS)-1 and SOCS-3 signalling.^16^ MerTK recognises the exposed phosphatidylserine on the surface of apoptotic cells, in association with its ligands (Gas-6 and Galectin-3), while their efferocytosis induces a monocyte/macrophage functional switch towards resolution–tissue repair responses.^17^

Secretory leucocyte protease inhibitor (SLPI) is a small protein (11.7 kDa) secreted by epithelial and myeloid cells that can suppress monocyte/macrophage proinflammatory responses through inhibition of NF-κB signalling.^18–21^ SLPI is shown to exert immune-modulatory activities during tissue inflammation in a variety of inflammatory diseases such as sepsis, asthma and cancer.^22^ We recently identified SLPI, secreted in the liver by biliary epithelial cells and hepatic macrophages, as a modulator of circulating monocyte function in human ALF.^11^ In this study, we used a combination of human and murine experimental models in order to investigate the role of MerTK during resolution following acute liver injury and examine how SLPI, as a proresolving mediator, governs this immunological response in ALF.

## Methods

### Patients

Patients with acetaminophen-induced ALF (AALF, n=23) and non-acetaminophen induced ALF (NAALF, n=9) were recruited to the study within 24 hours following admission to the Liver Intensive Care Unit of King's College Hospital (London, UK). Inpatients with chronic liver disease (CLD, n=10) and healthy volunteers (HC, n=15) served as pathological and healthy controls. Exclusion criteria: age <18 or >65 years, neoplasia and immunosuppressive therapy; patients were identified for emergency transplantation according to King's College Hospital (KCH) criteria.^23^ The study was approved by the National Research Ethics Service (NRES) Health Research Authority (12/LO/0167). All diseased (06/Q2708/11) and normal (06/Q2702/61) liver tissue and blood samples (04/Q2708/41) were obtained through the Liver Unit of Queen Elizabeth Hospital (Birmingham, UK) after local ethics committee approval and patient consent. Clinical, haematological and biochemical parameters were determined on a haematological analyser (Siemens Advia 2120, Berks, UK).

### Mice

All animal experiments were conducted with approval by the Home Office and local ethics committees (PPL 70/7578). B6.129-MerTK^tm1Gr1/J^ (Mer^−/−^) and wild-type (WT) mice with an identical background (B6.129SF2/J) were obtained from The Jackson Laboratory. WT and Mer^−/−^ mice were age-matched and sex-matched (male, 8–10-week-old) for the experiments. Mice fasted overnight received an intraperitoneal injection of APAP (300 mg/kg, Sigma-Aldrich, UK) or saline and were studied at several time points. *SLPI administration in APAP mice*. WT mice (male, 8–10-week-old, C57BL/6J) fasted overnight and received an intraperitoneal injection of APAP or recombinant human (rh)-SLPI (16.5 µg/kg) (R&D Systems, UK) or both based on the SLPI plasma levels (8 hours post APAP) (see online supplementary methods 1). Mice sacrificed at 24 or 48 hours received a second SLPI intraperitoneal injection at 8 hours, while mice sacrificed at 48 hours received a third SLPI intraperitoneal injection at 24 hours.

10.1136/gutjnl-2016-313615.supp1 supplementary data

### Flow cytometry

Human monocytes and liver-derived macrophages were phenotypically characterised using flow cytometry on a fluorescence-activated cell sorting (FACS) Canto II analyser (BD Biosciences, UK), and data were analysed with FlowJo 10.1 software (Treestar, Ashland, OR). Murine liver-derived macrophages were phenotypically characterised using flow cytometry on an LSR Fortessa analyser (BD Biosciences, UK), and data were analysed with Flowlogic 600.0A software (Inivai Technologies) (see online supplementary methods 2–4).

### Gene expression analysis

Gene expression analysis was performed using the NanoString nCounter GX Human Immunology V2 assay (NanoString Technologies, Seattle, Washington, USA) profiling 594 immunology-related genes on FACS-separated cells (see online supplementary methods 5). The differential gene expression among subsets was calculated and plotted as heat map using the nSolver Analysis Software V.3.0 (NanoString Technologies, Seattle, Washington, USA). Statistically relevant results are considered with p<0.05 and a fold-change of 50% higher or lower.

### Ultra-performance liquid chromatography tandem mass-spectrometry (UPLC-MS) of acetaminophen and metabolites in mouse plasma

Mouse plasma samples were analysed for acetaminophen (APAP) and five metabolites (APAP-glucuronide, APAP-sulfate, APAP-cysteinyl, APAP-glutathione and APAP-N-acetylcysteinyl), as detailed (see online supplementary methods 6). Samples, deproteinised via solvent precipitation, were analysed by reversed-phase gradient chromatography on an Acquity Ultra Performance Liquid Chromatography system (Waters Corporation, Manchester, UK) with selective detection via MS/MS, in positive electrospray ionisation mode, via a Waters Xevo tandem quadruple (TQ)-S mass spectrometer. Quantification of each compound was relative to an appropriate deuterated internal standard (see online supplementary methods 6).

### Tissue sampling and imaging

Human liver tissue was obtained from patients with ALF (n=14) undergoing orthotopic liver transplantation,^4^ diseased liver tissue from patients with CLD (n=10), while normal liver (NL, n=6) tissue was derived from hepatic resection margins of colorectal malignancies. For phenotyping of macrophages, mononuclear cells were freshly isolated from ALF (n=8), CLD (n=10) and NL (n=6) liver tissue. For immunohistochemistry (IHC), liver and mesenteric lymph node tissues were obtained from ALF (n=6) and hepatic resections, serving as pathological controls (n=4). Single/double epitope enzymatic IHC on formalin-fixed paraffin-embedded (FFPE) tissue was performed to assess the number of positive cells for MerTK, HLA-DR, CD163, MPO and TUNEL. Double epitope fluorescent IHC was used to demonstrate colocalisation by Nuance multispectral analysis and confocal microscopy (see online supplementary methods 7).

### Cell culture and functional assays

SLPI effects were determined on cells cultured with (rh)-SLPI (0 and 0.5 μg/mL) (R&D Systems, UK). Cells were analysed for their phenotype and lipopolysaccharide (LPS)-stimulated (100 ng/mL) cytokine levels using flow cytometry and ELISA. SLPI effects on (a) monocyte migration and efferocytosis and (b) neutrophil oxidative burst and extracellular trap (NET) formation were also assessed; for blocking experiments, culture supernatants were preincubated with anti-SLPI antibody (α-SLPI) (R&D Systems, UK)^11^ (see online supplementary methods 8–14).

### Statistical analysis

Data analysis and graphing were performed using GraphPad Prism 6 software (GraphPad Software, La Jolla California). Statistical significance was assessed with non-parametric analyses, and results are presented as median with IQR, unless otherwise specified in figure legends.

## Results

### Resolution-like MerTK+ monocytes and hepatic macrophages are expanded in ALF

**Figure 1:**
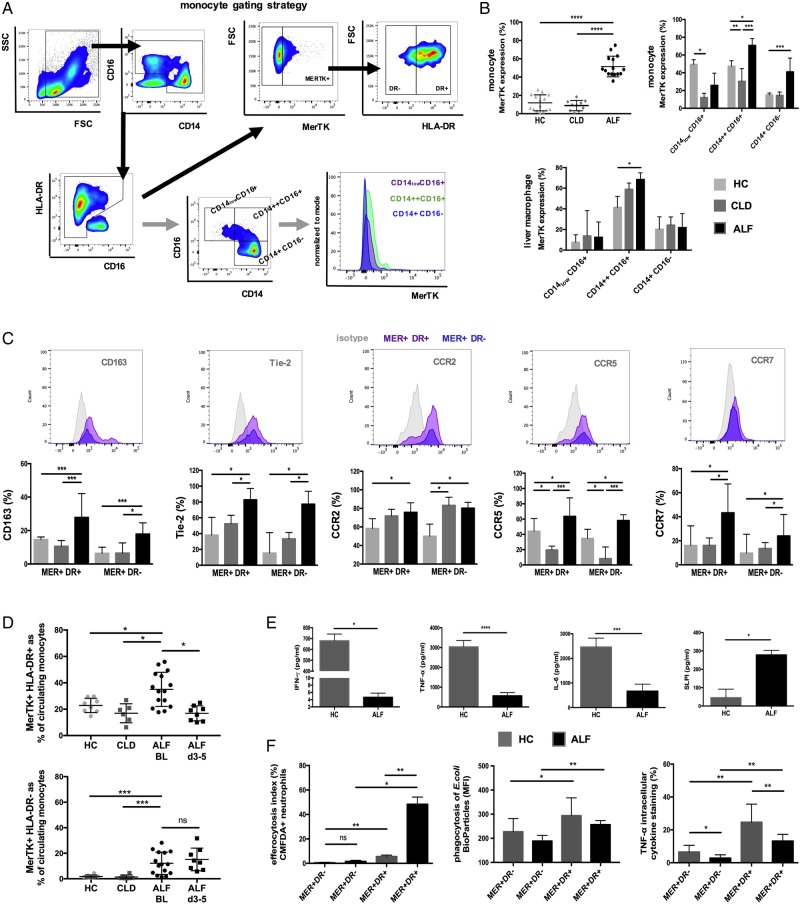
Characterisation of Mer tyrosine kinase (MerTK)+ circulating monocytes and liver-derived hepatic macrophages in patients with acute liver failure (ALF). (A) Flow cytometry analysis and gating strategy used to determine MerTK expression in circulating monocytes and their subsets. (B) Data show MerTK expression levels of (1) monocytes in patients with ALF (n=15), chronic liver disease (CLD) (n=10) and healthy controls (HC) (n=15), (2) liver-derived macrophages isolated from ALF (n=8), CLD (n=10) and normal liver (n=6) tissue. (C) Representative histograms and expression levels of monocyte surface markers in MerTK+HLA-DR± cells. (D) MerTK+HLA-DR+ and MerTK+HLA-DR- subsets as proportion (%) of circulating monocytes in HC (n=15), patients with CLD (n=10) and ALF on admission (n=15) and days 3–5 following their admission (n=8). (E) Inflammatory cytokine secretion in HC and ALF peripheral blood mononuclear cells (PBMC) supernatants (n=5 each) after microbial challenge (LPS 100 ng/mL, 6 hours), as determined by ELISA. (F) Proportion of MerTK+HLA-DR± monocytes that efferocytosed apoptotic neutrophils, phagocytosed *E. coli* bioparticles and produced tumour necrosis factor (TNF)-α after microbial challenge (n=5 each). Non-parametric (Mann-Whitney) statistical analysis was used. Data presented as median values with IQR. *p<0.05, **p<0.01, ***p<0.001, ****p<0.0001. SSC, side scatter; FSC, forward scatter.

Using flow cytometry, we assessed the phenotype of both circulating monocytes and hepatic macrophages, freshly isolated from human explant tissue, in ALF, CLD and HC. Patients with ALF exhibit a marked increase in the proportion of MerTK+ cells, when compared with HC and CLD (table 1 and figure 1A,B). Patients with acetaminophen-induced ALF (AALF) show a higher percentage of MerTK+ monocytes, physiological and biochemical indices of disease severity, compared with those with non-acetaminophen-induced ALF (NAALF) (table 2). MerTK+ cells correlated positively with SIRS score (r=0.47; p<0.01), AST (r=0.45; p<0.01), international normalised ratio (r=0.37; p<0.01), heart rate (r=0.36; p<0.05), and negatively with bilirubin (r=−0.4; p<0.05), mean arterial pressure (r=−0.49; p<0.01) and monocyte count (r=−0.47; p<0.01).

**Table 1:** Clinical and physiological characteristics of patients with acute liver failure (ALF) in comparison with CLD and HC groups

Parameter	ALF	CLD	HC
Number of patients	32	10	15
Age	35* [28–45]	52 [44–65]	29* [26–45]
Sex (M:F)	15:17	6:4	6:9
Aetiology	*Drug-induced* Acetaminophen [23] Mixed overdose [2] *Non-drug induced* Pregnancy-related [3] Budd-Chiari [1] Hepatitis B [1] Acute Wilsons [1] Ischaemia [1]	ALD [4] NAFLD [3] HFE [1] PBC [1] PSC [1]	n/a
WCC (×10^9^/L)	10.4* [6.7–15.5]	4.46 [3.7–5.1]	n/a
Monocytes (×10^9^/L)	0.22 [0.13–0.4]	0.31 [0.24–0.42]	n/a
Creatinine (μmol/L)	139.5** [90–242]	73 [56–79]	n/a
INR	5.4*** [2.8–8.8]	1.4 [1.15–1.6]	n/a
Bilirubin (μmol/L)	88*** [49.5–177]	49 [12–70]	n/a
AST (IU/mL)	5199*** [1155–8499]	45 [28–55]	n/a
Encephalopathy	2*** [1–3]	1 [0–1]	n/a
Child's Pugh	n/a	9 [8–11]	n/a
MELD	39.5* [33–40]	12 [7–19]	n/a
SOFA	13 [10–16]	n/a	n/a

*p=0.006; **p=0.001; ***p<0.0001, compared with CLD group.

ALD, alcoholic liver disease; AST, aspartate aminotransferase; CLD, chronic liver disease; HC, healthy contols; HFE, haemochromatosis; INR, international normalised ratio; MELD, Model for End Stage Liver Disease; NAFLD, non-alcoholic fatty liver disease; PBC, primary biliary cholangitis; PSC, primary sclerosing cholangitis; SOFA, sequential organ failure assessment; WCC, white (leucocyte) cell count.

**Table 2:** Clinical and physiological parameters of patients with acetaminophen-induced acute liver failure (AALF) and non-acetaminophen induced AALF (NAALF)

Parameter	AALF	NAALF
Number of patients	23	9
Number of patients transplanted/died	8/23	4/9
MERTK monocyte expression (%)	51.50** [40.75–73.75]	35.95 [31.45–52.25]
WCC (×10^9^/L)	9.57 [6.34–15.96]	11.4 [8.0–18.4]
Monocytes (×10^9^/L)	0.21** [0.14–0.36]	0.54 [0.31–0.98]
Creatinine (μmol/L)	148 [76.5–237.5]	193 [93–362]
INR	6.4** [3.0–8.4]	2.7 [1.4–4.6]
Bilirubin (μmol/L)	74 [48–96]	172 [42.5–587]
Lactate (mmol/L)	3.0 [2.2–6.8]	2.7 [1.8–4.0]
AST (IU/mL)	5172* [1500–7436]	2088 [432–6176]
Encephalopathy	2* [1–3]	1 [1–2]
SIRS score	2 [1–3]	2 [0–3]
APACHE II	19 [14–25.5]	20 [11–27]
SOFA	12 [10–15.5]	13 [10–16.5]
MELD	40 [33–40]	38 [24–40]

*p<0.05 and **p<0.01, compared with NAALF group.

APACHE II, Acute Physiology and Chronic Health Evaluation II score; AST, aspartate aminotransferase; INR, international normalised ratio; MELD, Model for End Stage Liver Disease; SIRS, systemic inflammatory response syndrome; SOFA, Sequential Organ Failure Assessment score; WCC, white (leucocyte) cell count.

In line with previous data,^24^ MerTK+ cells in ALF are characterised by a resolution-like HLA-DR^high^CD163^high^Tie-2^high^ immunophenotype compared with CLD and HC (figure 1C). Peak levels of MerTK+HLA-DR^high^ cells are detected on admission to the Liver Intensive Care Unit (figure 1D), which subsequently decline to levels similar to HC by day 3–5. In contrast to HC and CLD groups, MerTK+HLA-DR_low_ cells are detected and significantly elevated in circulatory and tissue comparments in patients with ALF and remain persistently elevated following their admission (figure 1D and see online supplementary figure S2A,B). Analyses of MerTK+ cells based upon their HLA-DR expression identify key differences in their functional profile. Compared with HC and MerTK+HLA-DR_low_ cells, MerTK+HLA-DR^high^ cells have an enhanced clearance of apoptotic (CMFDA+ Annexin-V^high^ neutrophils) and infective (pHrodo E. coli BioParticles) material, with attenuated secretion of proinflammatory (eg, tumour necrosis factor (TNF)-α) mediators following microbial challenge (figure 1E, F, and see online supplementary figure S1B).

### Gene expression profile of MerTK+ monocytes in ALF

**Figure 2:**
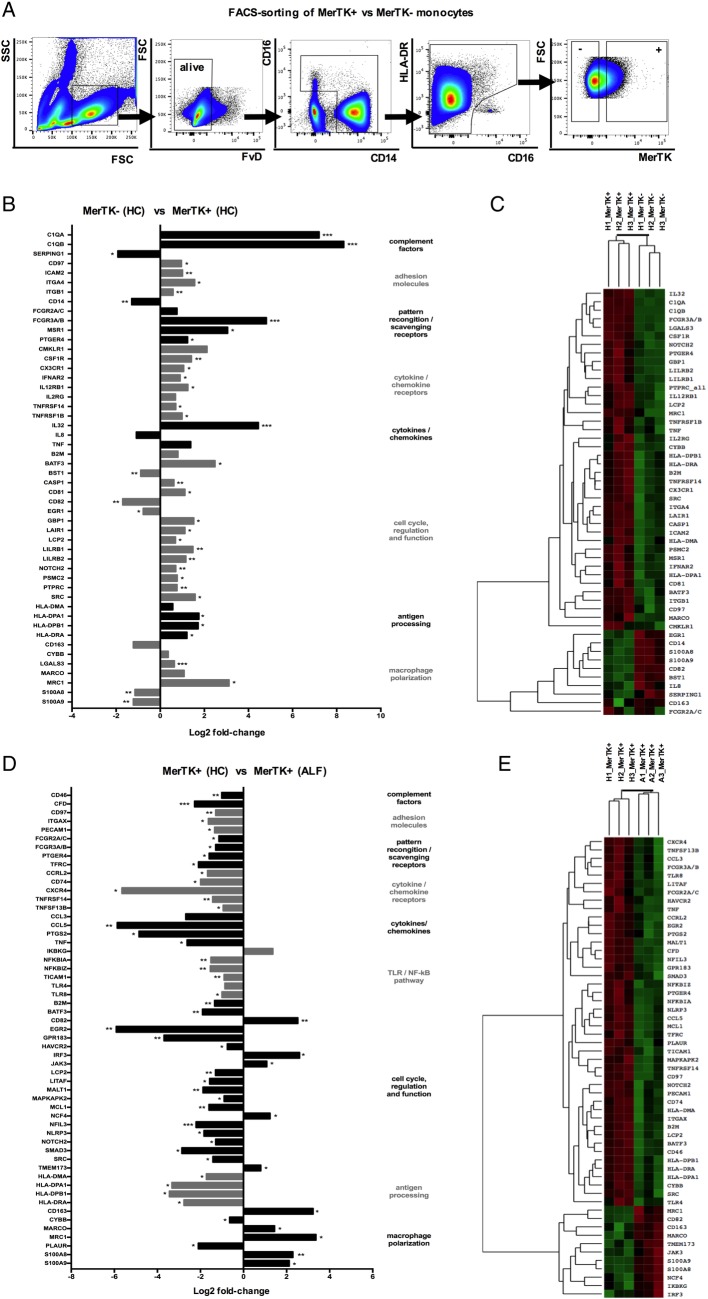
Gene expression pattern of Mer tyrosine kinase (MerTK)+ monocytes in heatlhy controls (HC) and acute liver failure (ALF). (A) Human MerTK+ and MerTK− monocytes were FACS-sorted in HC and patients with ALF (n=3 each), with a gating strategy displayed by representative flow cytometry plots. (B–E) Highly pure isolates of MerTK± subsets were subjected to quantitative microarray gene expression analysis (nCounter GX Human Immunology V2 kit, profiling 594 immunology-related human genes; NanoString Technologies, Seattle, Washington, USA). Data show the log2 fold-change of expression and agglomerative cluster (heatmap, z-score; green=min and red=max magnitude of expression) of 50 chosen differentially expressed genes, comparing MerTK+ versus MerTK− monocytes in HC (B and C), and MerTK+ monocytes in ALF versus MerTK+ monocytes in HC (D and E). *p<0.05, **p<0.01, ***p<0.001. SSC, side scatter; FSC, forward scatter.

To fully characterise the MerTK+ population in ALF, we performed FACS-sorting of MerTK± monocytes from HC and ALF (figure 2A) and subjected these cells to a quantitative gene expression array. Compared with MerTK−, MerTK+ monocytes at steady state (figure 2B, C), and in ALF (see online supplementary figure S2), have a transcriptional profile consistent with a more differentiated ‘tissue-like’ phenotype, characterised by a significant upregulation of genes responsible for adhesion (eg, ICAM2, ITGA4, ITGB1), phagocytosis/pattern-recognition (FCGR2A/C, FCGR3A/B, MSR1, C1q), cell proliferation/survival (eg, C81, LAIR1, SRC), antigen presentation (HLA-DPA1, HLA-DPB1, HLA-DRA) and macrophage M2-like polarisation (LGALS3, MARCO, MRC1, CMKLR1, CSF1R) (figure 2B, C and see online supplementary figure S2).

Comparison of the transcriptional profile between MerTK+ monocytes in ALF and HC (figure 2D, E) revealed striking differences in line with the phenotypic and functional readouts (figure 1A–F). MerTK+ monocytes in ALF exhibit a marked reduction in a number of regulatory pathways, notably in antigen-processing MHC class II associated transcripts (HLA-DMA, HLA-DPA1, HLA-DPB1, HLA-DRA), TLR/NF-kB-dependent proinflammatory pathway (NFKBIA, NFKBIZ, TICAM1, TLR4, TLR8), phagocytosis/pattern-recognition (FCGR2A/C, FCGR3A/B) and cytokines/chemokines (CCL5, PTGS2, TNF) (figure 2D, E). Moreover, these cells maintain an M2-like skewed profile (eg, CD163, MARCO, MRC1), activation of the downstream MerTK/cytokine signalling pathway (IRF3, JAK3) with a concomitant downregulation in genes linked with cell activation (NLRP3, SMAD3, SRC) (figure 2D, E).

### MerTK+ macrophages are important in the resolution of acute liver injury

**Figure 3:**
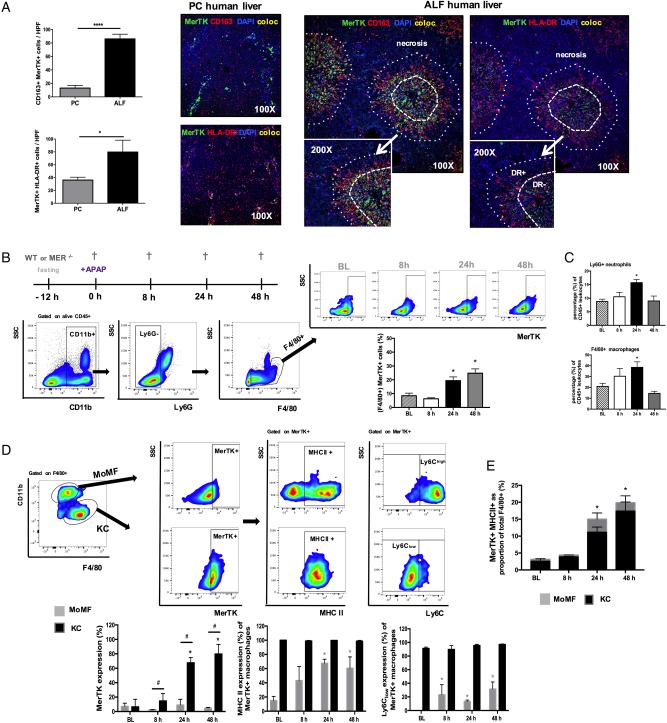
Mer tyrosine kinase (MerTK)+ hepatic macrophages are expanded in human and experimental APAP-induced acute liver injury. (A) Representative confocal images for MerTK (green), CD163 (red), HLA-DR (red), DAPI (blue) and colocalisation (yellow) in pathological control (n=4) and acute liver failure (ALF) (n=6) human liver tissue (100×, inset 200×). Data show enumeration of CD163+MerTK+ and MerTK+HLA-DR+ cells in centrilobular areas of pathological control (PC, n=4) and ALF (n=6) liver. **(**B–E) Wild-type (WT) mice dosed with APAP were studied at 8, 24 and 48 hours, while untreated mice served as baseline controls (n=4/group). (B) Schematic of experimental dosing, representative flow cytometry analysis and gating strategy used to identify F4/80+ hepatic macrophages and determine their MerTK expression levels. (C) Ly6G+ neutrophils and F4/80+ macrophages as percentage (%) of total liver CD45+ leucocytes, as determined by flow cytometry. (D) Representative flow cytometry gating strategy used to identify (CD11b_low_F4/80^high^)-resident Kupffer cells (KC) and (CD11b^high^F4/80_low_) monocyte-derived macrophages (MoMF) to determine their MerTK expression. Further subanalysis examined the MHC class II and Ly6C expression levels of MerTK+ macrophages in both MoMF (grey bars) and KC (black bars) subpopulations. (E) Data show MerTK+MHCII+ macrophages as proportion of total F4/80+ cells and the relative contribution of MoMF (grey) or KC (black). Non-parametric (Mann-Whitney) statistical analysis was used. Data are presented as median values with IQR. * or ^#^p<0.05, **p<0.01, ****p<0.0001.

We employed multispectral and confocal imaging to delineate the topography and phenotype of MerTK+ macrophages in human ALF liver tissue. Compared with pathological controls, we confirm a numerical increase of MerTK+ cells, of monocyte/macrophage lineage (CD163+), that localise to areas of centrilobular necrosis in the ALF liver (figure 3A and see online supplementary figure S3A). In non-acutely inflamed tissue, the proportion of MerTK+ cells derived from infiltrating monocytes (MAC387+MerTK+) was <39%. Interestingly, the proportion of these circulatory derived cells was similar in ALF explant tissue (figure 3A and see online supplementary figure S3C). Similar to other models of sterile liver injury,^25^ ^26^ we show that these avidly phagocytic (figure 1F) MerTK+HLA-DR^high^ macrophages infiltrate and form ring-like structures around necrotic areas in the ALF liver (figure 3A).

Given our data demonstrating a marked expansion of MerTK+ cells in human ALF, with resolution-like properties, we sought to determine their biological relevance in acute liver injury, using APAP-treated WT and Mer-deficient (Mer^−/−^) mice. First, by applying UPLC-MS on plasma derived from both groups (baseline vs 8 hours post APAP), we show that WT and Mer^−/−^ mice have no differences in APAP metabolite concentrations, indicating that the Mer^−/−^ do not differ in their APAP metabolism (see online supplementary figure S4). Next, we examined the temporal expression and immunophenotype of MerTK+ hepatic macrophages in WT mice (figure 3B, E). Here, we identify an increase in the overall proportion of F4/80+CD11b+Ly6G− MerTK+ macrophages at 24 and 48 hours after APAP administration (figure 3B). Using established gating strategies,^7^ ^10^ ^27^ we demonstrate that the increase in MerTK+ cells is predominantly on the resident KC population (figure 3D). Compared with MoMF, MerTK+ KC are characterised by a MHCclassII^high^ Ly6C_low_ expression profile (figure 3D) which is associated with highly phagocytic^14^ ^28^ and prorestorative capabilities^27^ ^29^ and bears striking similarities to the MerTK+HLA-DR^high^ phenotype observed in patients with ALF (figure 1D). Thus, KC greatly contribute to the increased proportion of MerTK+MHCclassII^high^ macrophages detected at 24 and 48 hours following APAP treatment (figure 3E).

**Figure 4:**
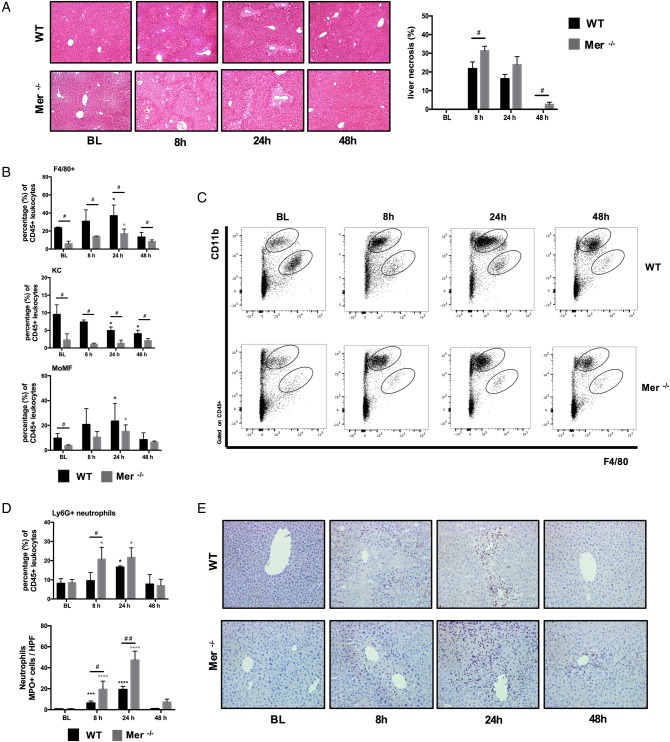
Mer tyrosine kinase (MerTK)-deficient mice are characterised by increased hepatic inflammation and reduced macrophages following APAP-induced liver injury. Wild-type (WT) (black bars) and Mer^−/−^ (grey bars) mice dosed with APAP were studied at 8, 24 and 48 hours, and untreated mice served as baseline controls (n=4/group). (A) Representative images of H&E stained liver tissue and quantification of necrotic area (%). (B) F4/80+ hepatic macrophages, Kupffer cells (KC) and monocyte-derived macrophages (MoMF) as percentage of total liver CD45+ leucocytes, as determined by flow cytometry. (C) Representative flow cytometric analysis from liver CD45+ leucocytes showing detection of (CD11b^high^F4/80_low_) MoMF and (CD11b_low_F4/80^high^) KC. (D) Ly6G+ hepatic neutrophils as percentage of total liver CD45+ leucocytes and enumeration of MPO+ neutrophils using flow cytometry and immunohistochemistry, respectively. (E) Representative images of liver tissue stained for MPO+ (purple) cells (200×). Non-parametric (Mann-Whitney) statistical analysis was used. Data are presented as median values with IQR. * or ^#^p<0.05, ***p<0.001, ****p<0.0001.

However, APAP administration in Mer^−/−^ mice resulted in a significantly higher and persistent degree of acute liver injury, compared with WT mice (figure 4A). Mer^−/−^ mice had a significantly lower proportion of F4/80+ hepatic macrophages at steady state and following APAP dosing (figure 4B) that was attributable to a depletion in the proportion of MHCclassII^high^Ly6C_low_ expressing resident KCs (figure 4B, C and see online supplementary figure S1D). In keeping with its role for neutrophil homeostasis and clearance,^30^ mice lacking MerTK displayed a significantly higher number of activated (MPO+) and proportion of (Ly6G+) hepatic neutrophils at peak (8 hours) and resolution phases (24 hours) of APAP-induced liver injury, when compared with WT mice (figure 4D, E).

### MerTK+ macrophages can be induced by the microenvironmental mediator SLPI in vitro

**Figure 5:**
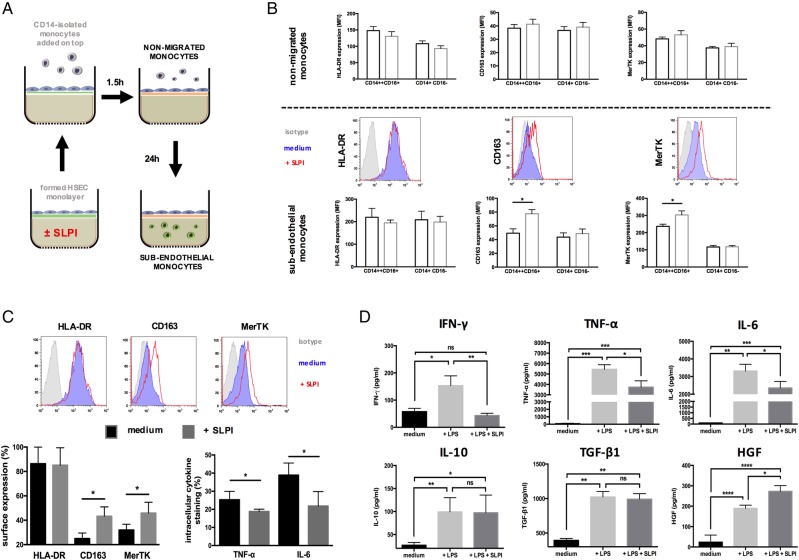
Secretory leucocyte protease inhibitor (SLPI) induces a Mer tyrosine kinase (MerTK)^high^HLA-DR^high^ phenotype in monocytes and liver-derived macrophages. (A–B) Effects of recombinant human (rh)-SLPI (0 and 0.5 µg/mL) on monocyte migration across hepatic endothelium were determined (n=3 independent experiments). (A) Schematic of migration assay: CD14-isolated monocytes are added on top of a preformed hepatic endothelium monolayer; non-migrated monocytes are harvested 1.5 hours after, while subendothelial monocytes are obtained 24 hours later. Phenotypic characterisation of non-migrated and subendothelial monocytes was determined by flow cytometry. (B) Data show HLA-DR, CD163 and MerTK expression levels and representative histograms (CD14++CD16+ subset) for (top panel) non-migrated and (lower panel) subendothelial monocytes. Results expressed as mean fluorescence intensity (MFI). (C and D) Effects of (rh)-SLPI (0 and 0.5 µg/mL) on hepatic macrophages isolated from normal liver explant tissue were assessed (n=5). (C) Data show representative histograms and surface marker expression in the CD14++CD16+ subset and intracellular cytokine levels in total monocytes following microbial challenge (LPS 100 ng/mL). (D) LPS-stimulated inflammatory cytokine levels (pg/mL) in hepatic mononuclear cell culture supernatants, as determined by ELISA. Non-parametric (Mann-Whitney) statistical analysis was used. Data presented as median values with IQR. *p<0.05, **p<0.01, ***p<0.001, ****p<0.0001. IFN, interferon; IL, interleukin; iso, isotype control antibody; ns, non-significant; TNF, tumour necrosis factor.

Given the marked upregulation of MerTK within the ALF liver, we sought to examine whether the key microenvironmental mediator SLPI,^11^ released by monocytes/macrophages^11^ in ALF (figure 1E), could be responsible for its induction. We show that SLPI induces MerTK+HLA-DR^high^ monocytes following their transmigration across activated hepatic endothelium^31^ (figure 5A, B), a phenotype that is also recapitulated in human liver-derived hepatic macrophages (figure 5C). Importantly, these cells exhibit augmented secretion of anti-inflammatory/proresolving (interleukin (IL)-10, transforming growth factor (TGF)-β1, hepatocyte growth factor (HGF)) mediators but attenuated levels of proinflammatory factors (interferon (IFN)-γ, TNF-α, IL-6) (figure 5D).

### SLPI-induced MerTK+ macrophages suppress neutrophil activation and promote their clearance

**Figure 6:**
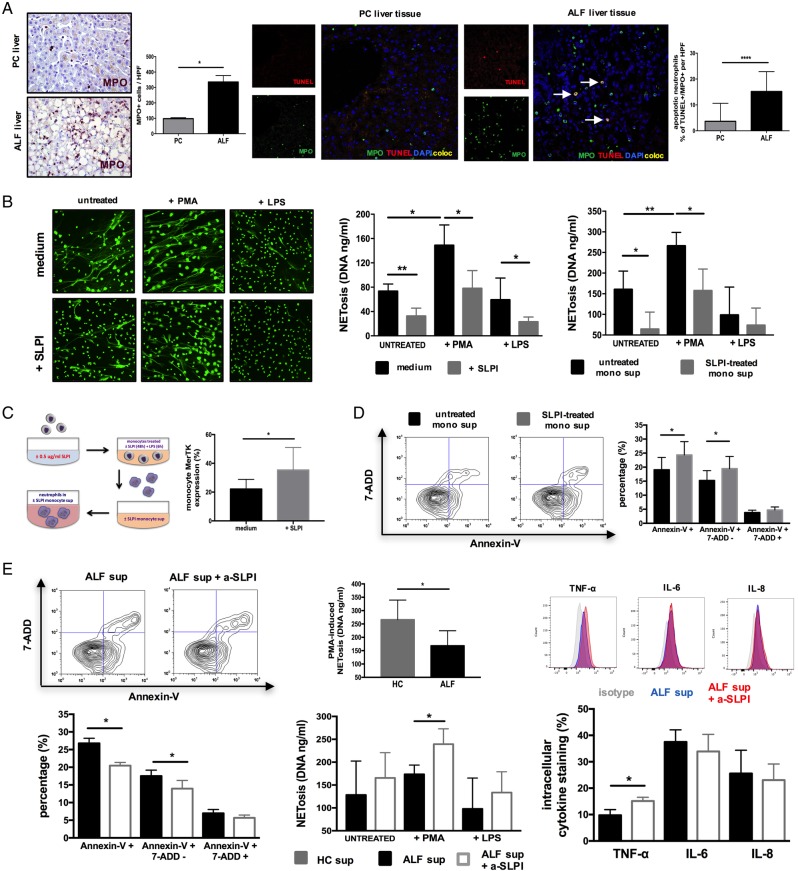
Secretory leucocyte protease inhibitor (SLPI) suppresses neutrophils through Mer tyrosine kinase (MerTK)^high^HLA-DR^high^ monocytes in a paracrine manner. (A) Representative immunohistochemistry (IHC) (left) and confocal (right) micrographs and enumeration of (MPO+) hepatic neutrophils and (MPO+TUNEL+) apoptotic neutrophils in centrilobular areas of pathological control (PC, n=4) and acute liver failure (ALF) (n=6) human liver tissue. IHC images (400×) show MPO+ (purple) cells. Confocal images show MPO (green), TUNEL (red), DAPI (blue) and coexpression (yellow) (400×). (B–E) Autocrine and paracrine effects of recombinant human (rh)-SLPI (0 and 0.5 μg/mL) on neutrophils were examined (n=5). (B) Neutrophil extracellular trap formation (NETosis) was determined fluorometrically (DNA, ng/mL) in culture following stimulation with PMA (25nM) or LPS (100 ng/mL). (Left) Representative images of NETs using SYTOX Green Dye (1 μM). (Right) Data show NETosis (DNA, ng/mL) quantified in culture (1) with/without SLPI and (2) with ±SLPI-treated monocyte culture supernatants (n=3). (C) Paracrine experimental approach for neutrophils and data showing monocyte MerTK expression levels after culture with/without SLPI (48 hours). (D) Representative Annexin-V/7-AAD staining and percentage of apoptotic neutrophils in culture with ±SLPI-treated monocyte supernatants (n=5). (E) SLPI paracrine effects were assessed by blocking SLPI's activity on monocytes in ALF plasma (α-SLPI, 5 µg/mL) (n=5). (Left) Representative Annexin-V/7-AAD staining and percentage of apoptotic neutrophils; (middle) NET formation (DNA, ng/mL) and (right) LPS-stimulated (100 ng/mL) intracellular cytokine levels of neutrophils (n=5 each). Data presented as median values with IQR. *p<0.05, **p<0.01, ****p<0.0001. HC, healthy controls; IL, interleukin; ns, non-significant; TNF, tumour necrosis factor.

Having detected a higher number of apoptotic neutrophils in the ALF liver (figure 6A), we performed a series of experiments to determine whether SLPI can also modulate neutrophil activation and their subsequent clearance. We confirm that SLPI does not directly alter neutrophil survival, TLR-evoked inflammatory responses and reactive oxygen species production (see online supplementary figure 5A,B). However, in line with recent reports,^32^ SLPI directly (at physiological concentrations detected in ALF) reduced the formation of neutrophil extracellular traps (NETosis) following stimulation with either phorbol myristate acetate (PMA) or LPS (figure 6B). To evaluate whether MerTK^high^ cells modulate neutrophil function in a paracrine manner (figure 6C), we assessed the effects of soluble mediators released from healthy SLPI-treated and ALF monocytes on neutrophils (figure 6B, D). Neutrophils cultured in supernatants derived from SLPI-treated and ALF monocytes had a significantly reduced survival and PMA-induced NETosis (figure 6B, D). To determine whether the elevated SLPI levels in ALF^11^ could account for that, we repeated these experiments following SLPI blockade (α-SLPI). Inhibition of SLPI's activity in ALF-derived monocyte supernatants restored neutrophil survival, LPS-stimulated secretion (TNF-α) and increased PMA-evoked NETosis (figure 6E).

**Figure 7:**
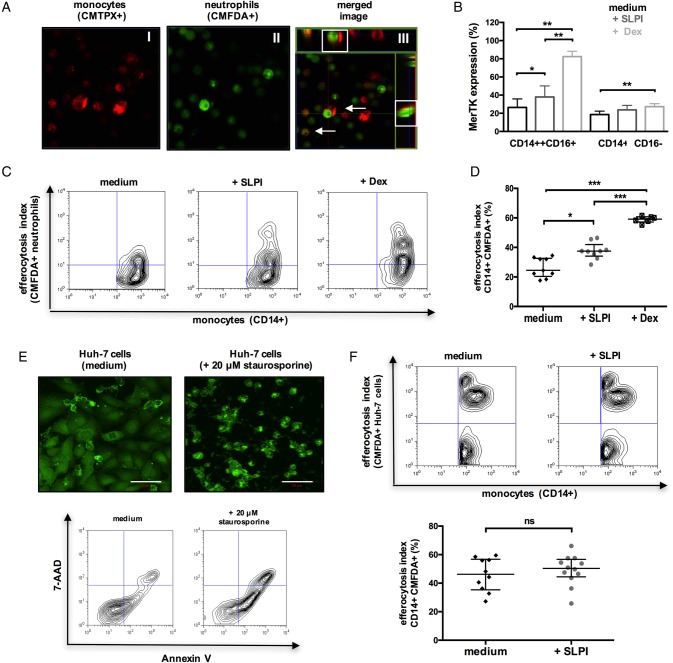
Secretory leucocyte protease inhibitor (SLPI) enhances the monocyte clearance of apoptotic neutrophils. (A–D) CD14-isolated monocytes were cultured with medium or (rh)-SLPI (0.5 μg/mL) or dexamethasone (100 nM) for 48 hours and then coincubated (4 hours) with apoptotic neutrophils (n=3 independent experiments). (A) Representative confocal microscopy images of CMTPX-labelled monocyte engulfment of apoptotic CMFDA-labelled neutrophils (original magnification ×63); merge/z-stack images: arrows showing colocalised/engulfed cells. (B) Data show Mer tyrosine kinase (MerTK) expression in monocyte subsets after culture (48 hours) with different treatments. (C and D) Representative flow cytometry plots and percentage of monocytes that phagocytosed CMFDA-labelled neutrophils. (E and F) CD14-isolated monocytes were cultured with medium or (rh)-SLPI (0.5 μg/mL) for 48 hours and then coincubated (4 hours) with apoptotic Huh-7 hepatoma cells (n=3 independent experiments). (E) (Upper) Representative confocal microscopy images of CMFDA-labelled Huh-7 cells and (lower) representative Annexin-V/7-AAD staining of Huh-7 cells treated with/without 20 μM STS (50 μm, scale bars). (F) Representative flow cytometry analysis and percentage of monocytes that phagocytosed CMFDA-labelled (STS-treated) apoptotic Huh-7 cells. Non-parametric (Mann-Whitney) statistical analysis was used. Data are expressed as median values with IQR. *p<0.05, **p<0.01, ****p<0.0001. ns, non-significant.

In addition to suppressing neutrophil activation, SLPI also augments their clearance. Similar to MerTK+HLA-DR^high^ cells in patients with ALF (figure 1F), both SLPI-treated and dexamethasone-treated (positive control)^33^ monocytes exhibited enhanced uptake of apoptotic neutrophils (efferocytosis) (figure 7A, D), but not hepatocytes, when compared with untreated cells (figure 7E-F). Together, we identify that SLPI fulfils the criteria as a proresolving mediator^34^ in ALF through the induction of a MerTK+HLA-DR^high^ phenotype which (a) counter-regulates the production of proinflammatory cytokines while promoting resolution/tissue-repair mediator release, (b) suppresses neutrophil activation and NET formation, (c) induces neutrophil apoptosis and enhances their subsequent clearance.

### SLPI induces MerTK+ macrophages in vivo and promotes resolution following acute liver injury

**Figure 8:**
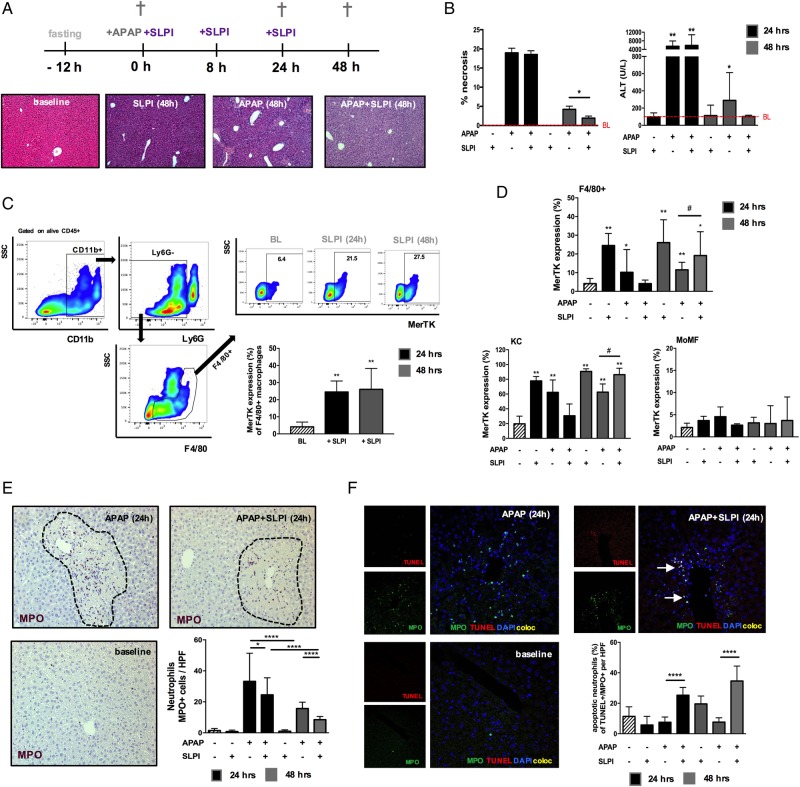
Secretory Leucocyte protease inhibitor (SLPI) administration in wild-type (WT) mice induces Mer tyrosine kinase (MerTK)+ macrophages and promotes hepatic resolution following APAP-induced acute liver injury. WT mice were dosed with SLPI (−/+) or APAP (+/−) or APAP plus SLPI (+/+) while untreated mice (−/−) served as baseline controls (n=6/group). Mice were studied at baseline (white bars), 24 hours (black bars) and 48 hours (grey bars). (A) Schematic describes the experimental dosing and representative images of H&E-stained livers (baseline and 48 hours). (B) Quantification of necrosis and plasma alanine transaminase (ALT) levels. (C) Representative flow cytometry analysis and data show MerTK expression of F4/80+ macrophages at baseline and following SLPI administration. (D) Data show MerTK expression levels of F4/80+ macrophages, subanalysed into (CD11b_low_F4/80^high^)-resident Kupffer cells (KC) and (CD11b^high^F4/80_low_) monocyte-derived macrophages (MoMF). (E) Representative liver immunohistochemistry images at baseline and 24 hours (n=5 each) and enumeration of MPO+ (purple) hepatic neutrophils (200×). (F) Representative liver confocal micrographs at baseline and 24 hours (n=5 each) stained for MPO (green), TUNEL (red), DAPI (blue); coexpression (yellow) (400×). Data show the percentage of (MPO+TUNEL+) apoptotic neutrophils. Non-parametric (Mann-Whitney) statistical analysis was used. Data are presented as median values with IQR. * or #p<0.05, **p<0.01, **** p<0.0001. SSC, side scatter.

We determined whether prorestorative MerTK+ macrophages could be induced by exogenous SLPI administration in mice using the experimental model of ALF. We found comparable biochemical and histological indices of liver injury at 24 hours between APAP and APAP+SLPI-treated mice (figure 8A, B). However, SLPI administration in APAP mice led to a significant reduction in those indices during the resolution phase (48-hour timepoint) of APAP-induced injury (figure 8A, B). To examine whether SLPI induces resolution-like macrophages in vivo, as described in vitro (figure 5A–C), we determined the MerTK expression levels of (F4/80+) hepatic macrophages at steady state and during APAP-induced acute liver injury with/without SLPI administration (figure 8C, D). Here, we show that SLPI induces an increase in the (F4/80+) overall proportion of MerTK+ macrophages at steady state (figure 8C) and at 48 hours post APAP dose (figure 8D). Furthermore, the increase in MerTK expressing macrophages following SLPI treatment is confined to the resident KC population (figure 8D).

In view of the data identifying the mechanisms by which SLPI promotes neutrophil death and clearance, we also examined the number of apoptotic neutrophils in APAP mice using immunohistochemistry and confocal imaging. SLPI-treated APAP mice had a significantly lower number of MPO+ cells and higher proportion of apoptotic neutrophils (MPO+TUNEL+) at 24 and 48 hours, when compared with APAP mice (figure 8E, F). Taken together, our results reveal that during APAP-induced liver injury in mice, SLPI administration promotes resolution responses by imprinting a resolution-like, MerTK+, hepatic macrophage phenotype and modulates neutrophil activation and survival.

## Discussion

We describe an expansion of monocytes and hepatic macrophages that exhibit an immune-regulatory MerTK+HLA-DR^high^ phenotype with resolution-like characteristics, detected in both circulatory and tissue compartments of patients with ALF. We also show that these cells infiltrate the inflamed liver and form ring-like structures around areas of hepatic necrosis. In line with other models of acute tissue injury,^24^ ^35–37^ functional analyses of these cells reveal that they possess suppressed innate immune but enhanced resolution responses, typified by augmented clearance of cellular/infective material and secretion of anti-inflammatory/tissue-repair mediators (eg, SLPI) but reduced proinflammatory cytokines (eg, TNF-α). In line with previous data,^24^ comparison of gene expression profiles between MerTK+ and MerTK− monocytes in steady state and ALF reveal that they possess a more differentiated lineage ‘tissue-like’ profile, with increased expression of a number of genes associated with innate and adaptive effector functions.

Our data demonstrate marked elevations in circulating levels of MerTK+ cells in patients with both acetaminophen-induced and non-acetaminophen induced ALF, with highest MerTK levels detected in patients with AALF with a greater severity of acute liver injury and adverse clinical outcome. Peak circulating MerTK levels are detected early on admission to our unit, which may reflect the extent of hepatic tissue resolution responses following acute hepatocellular necrosis. Future prospective studies are warranted to assses the utility of circulating MerTK levels as an early mechanistic biomarker of resolution responses and outcome in patients with acute liver injury.

Our data from the experimental model for this disease are first to provide novel insights into the origin and biological relevance of these regulatory cells during acute hepatic injury. Here, we show that the proportion of MerTK-expressing cells are specifically increased in the resident KC, and not infiltrating macrophage, population following APAP-induced acute liver injury. Moreover, MerTK+ KCs bear a MHCclass II^high^Ly6C_low_ phenotype which is the dominant phagocytic^14^ ^28^ and prorestorative^27^ ^29^ hepatic macrophage population and bears striking similarities with the ‘prophagocytic’ MerTK+HLA-DR^high^ cells in human ALF. Furthermore, while the proportion of monocyte-derived macrophages expressing MerTK does not substantially increase in acute liver injury, these cells also acquire the MHCclassII^high^Ly6C_low_ phenotype, in line with their role in resolving inflammation.^6^ ^7^

We demonstrate that Mer-deficient mice have a reduced proportion of resident KCs prior to and following APAP administration. In view of its role in promoting cell survival through activation of antiapoptotic pathways,^38^ these data suggest that activation of MerTK in KCs is of importance in differentiation and restoration following acute liver injury. Further studies are warranted to identify the precise mechanisms through which MerTK regulates this process in both human and experimental models of disease.

Our findings in APAP-treated Mer-deficient mice echo recent data showing that MerTK promotes resolution responses by dampening innate responses and augmenting clearance of neutrophils following acute tissue injury.^30^ ^39^ ^40^ In the absence of MerTK, we report a significant depletion in hepatoprotective resident macrophages^12^ ^13^ (MHCclass II^high^Ly6C_low_) with the highest phagocytic capabilities,^28^ a reciprocal increase in hepatic neutrophils and persistent necrosis following APAP administration. Taken together, these data indicate that the MerTK-bearing cells identify a hepatic macrophage population with enhanced phagocytic capabilities that evolve following acute hepatocellular necrosis serving to drive hepatic resolution responses.

In both experimental and human models of ALF, we identify SLPI as a microenvironmental mediator that critically regulates the interplay between myeloid cells to promote hepatic resolution responses through the induction of a MerTK^high^ phenotype. Specifically, we show that SLPI selectively induces MerTK expression in the resident KCs while concomitantly increasing the number of apoptotic neutrophils. However, there are no data on how SLPI modulates neutrophil function. Here, we demonstrate that SLPI induces neutrophil apoptosis in a paracrine manner and augments their subsequent clearance through MerTK^high^ cells. The effect of SLPI on cell clearance appears to be highly selective in view of the fact that it does not alter clearance of APAP-treated apoptotic or necrotic parenchymal cells. Furthermore, we determined that SLPI can also drive proresolution responses through attenuation of NET formation, a process that has been shown to exacerbate acute tissue damage through activation of the inflammasome in ischaemia–reperfusion injury.^41^ Further studies are required to examine the role of NETosis in modulating myeloid cell activation and its effects on acute liver injury.

Although anti-inflammatory programmes initiated following apoptotic cell uptake are beneficial in resolving tissue injury, they have the undesirable potential to dampen antimicrobial responses.^25^ Our data support this notion as we demonstrate that acute liver injury reprogrammes myeloid cells towards MerTK-dependent resolution responses, which quell tissue inflammation and promote the clearance of debris, while at the same time suppress antimicrobial responses. Indeed, comparisons of MerTK transcriptional and functional profiles between ALF and healthy controls reveal striking differences. Here, MerTK+ monocytes in ALF have significant reductions in immune pathways associated with innate immune recognition, signalling, antigen presentation with a concomitant upregulation of M2-like and downstream MerTK signalling pathways. Taken together, we propose that circulating monocytes are specifically reprogrammed during ALF following activation of MerTK-dependent resolution responses.^4^ ^24^ ^42^ Further research is required to delineate the precise mechanisms of monocyte reprogramming in response to overwhelming tissue injury.

The functional ‘reorientation’ of myeloid cells is likely to be of pathogenic relevance in ALF, a condition that is characterised by systemic immune dysregulation, immuneparesis and a marked susceptibility to secondary infections.^4^ ^11^ Here, we also detect expanded numbers of MerTK+HLA-DR− cells, an immune cell subset with impaired innate responses to microbial challenge, during the evolution of human ALF. Given their enhanced lymph node and tissue homing receptor expression, it is tempting to speculate that these cells are generated following reprogramming within the inflamed liver and subsequently home to extrahepatic and circulatory compartments where they serve to suppress antimicrobial responses.^43^ Further work is required to delineate the recruitment and subsequent fate of MerTK-expressing myeloid cells during acute liver injury.

This work identified the induction of a prorestorative MerTK-positive cell subset in ALF that may promote tissue-repair responses, with implications for therapeutic intervention, where enhancing the local function of these cells could promote liver repair/regeneration. In this study, we used the proresolving actions of SLPI as proof-of-principle in order to highlight the biological significance of the MerTK+ phenotype in promoting resolution responses following acute liver injury. Using targeted strategies to harness the prorestorative capabilities of MerTK+ cells represents a promising therapeutic avenue in promoting tissue repair potentially in a number of acute hepatic inflammatory disorders. However, caution would need to be exercised when considering any of these therapeutic approaches, given the evidence of peripheral monocyte suppression in ALF,^11^ ^42^ indicating that the timing of therapy would need to be carefully calibrated in order to promote resolution while minimising the impact on immuneparesis. The balance of these biological processes must be rationalised when considering interventional strategies that promote liver repair processes while not exacerbating the risk of infection.

In conclusion, our data describe a marked expansion of a prorestorative MerTK+ phenotype in circulating monocytes and tissue-resident macrophages in ALF. These immunoregulatory cells evolve in response to acute liver injury and represent a novel immunotherapeutic target in acute hepatic injury to quell tissue-destructive responses and promote resolution. Furthermore, we show that SLPI acts as a key mediator in regulating the function of hepatic myeloid cells, to promote resolution responses through induction of MerTK expression within the resident macrophage population.
