# Supplementary material for: MerTK expressing hepatic macrophages promote the resolution of inflammation in acute liver failure
Source: Gut. 2017 Apr 27;67(2):333–47. doi: 10.1136/gutjnl-2016-313615 (PMC5868289; doi:10.1136/gutjnl-2016-313615)
Supplement: supplementary data [file gutjnl-2016-313615supp001.pdf]

Supplementary Methods

1. Acetaminophen (APAP) induced acute liver injury in mice.

Sample collection**.** Groups of mice were culled at each of the 6 time points after acetaminophen (paracetamol) dose [0, 8, 24, 48, 72 hours and 5 days; (n=5 each)]. Mice were placed under terminal anaesthesia with an intra-peritoneal injection of 0.15 ml of 20% pentobarbitone sodium. Following the confirmation of deep anaesthesia, a terminal blood sample was collected from the right ventricle. The left ventricle was catheterized, an incision was made in the right ventricle and PBS was perfused through the circulation at a rate of approximately 8 ml/minute. Visual inspection confirmed blanching of the liver and adequate perfusion. The liver was excised; the median lobe and gallbladder were separated from the rest of the liver and divided. Half the median lobe was place in formalin for fixation and half was snap frozen in liquid nitrogen. The remaining liver was roughly minced and placed on ice. ***Biochemical parameters****.* Blood was collected via cardiac puncture under terminal anesthesia with Pentoject (Centaur Services, UK) and liver function was assessed by measurement of plasma levels of alanine transaminase, total bilirubin and albumin by an AU680 chemistry analyzer (Beckman Coulter, UK). ***SLPI protein levels****.* SLPI levels were measured in plasma and liver homogenates using ELISA (USCN Life Sciences Murine SLPI ELISA, E91312Mu). ***SLPI mRNA levels by RT-PCR.*** RNA was extracted from snap frozen liver tissue using TRIzol® reagent (Life Technologies, Carlsbad, CA); 1 µg of RNA was reverse transcribed using Quantitect RT Kit (Qiagen, Dusseldorf, Germany). RT-PCR was performed using Taqman assays for murine SLPI (Mm00441530_g1) and B2M (Mm00437762_m1) as reference gene. Expression was compared using a ΔΔCT method.

***H&E staining**. Liver 4* μm-thick sections were stained with haematoxylin and eosin using the following protocol: dewaxing (10 min), rehydration (3 consecutive Et-OH baths with decreasing concentrations followed by a 10 min immersion in distilled water), Harris haematoxylin (5 min), washing (3 min in tap water), acid alcohol (rinse), washing in tap water (3 min), eosin (5 min), washing in tap water (3 min), dehydration (3 different 100% ethanol baths – rinsing), clarification (10 min in xylene), mounting with DPX.

Quantification of necrosis. For assessment of liver parenchymal injury, H&E stained sections from FFPE liver were imaged and assessment was carried out using ImageJ software. Fifteen low power fields (100X) completely filled with liver were selected, avoiding overlapping and large vessels as much as possible. All micrographs were saved as *jpeg.* files and randomized. The software was calibrated using a micrograph of a calibration slide taken with the 10× objective. At this magnification and image quality 1mm = 1082.0809 pixels giving each image a field area of 1,236,383.191 μm^2. Large vessels and non-liver parenchyma were hand selected and their area measured using the *Analyze → Measure* tool. By subtracting this value from the 100× field area we obtained the surface of the liver parenchyma. Then areas of necrosis were hand selected and measured, again by using the *Analyze → Measure* tool. The measurement was recorded in an Excel spreadsheet. The *Edit → Clear Outside* tool was used to exclude the non-necrotic liver tissue. Any large vessels or non-liver parenchyma areas within the remaining necrotic areas were again hand selected and measured. Measurements were recorded in the same spreadsheet. The area of necrosis in each field was calculated as the difference between the two recorded measurements. Necrosis was then expressed as percentage within liver parenchyma, on 15 consecutive low power fields (100×) in all groups, using the ImageJ software (Bethesda, Maryland, USA). The stains were analysed by a liver histopathologist (A.Q.) who was blinded to the

experimental data. Micrographs were obtained using a Leica DMR microscope equipped with a Leica DFC300 FX digital camera (Leica Biosystems, UK).

2. Isolation of human circulating and hepatic mononuclear cells.

Whole blood was used to isolate PBMCs (Ficoll-Paque Plus, GE Healthcare, UK) and neutrophils (Polymorphprep, Axis-Shield, Norway) by density gradient centrifugation. CD14+ monocytes were isolated from PBMCs using CD14+ magnetic beads (Miltenyi Biotec, UK), according to manufacturer's instructions. Cell viability was >95% according to trypan blue staining. Hepatic mononuclear cells were isolated from approximately 120-150 g of human liver tissue. Tissue was washed in cold sterile phosphate-buffered saline and was cut into small pieces in order to be mechanically digested in RPMI-1640 medium using a Stomacher400 Circulator (Cole-Parmer Instrument, United Kingdom) for 5 min at 260 speed. The tissue homogenate was then filtered and hepatic mononuclear cells were purified f by density gradient centrifugation using Lympholyte (Cedarlane Laboratories, Canada) for 20 min at 550x*g*. Hepatic mononuclear cells were washed in phosphate-buffered saline and were further examined for their phenotype and function.

3. Flow cytometry of murine hepatic mononuclear cells.

Liver tissue was perfused in situ via the left ventricle with PBS. Following excision of the liver, the tissue was mechanically dissociated and passed through 100 μm cell strainer. Cells were centrifuged at 60x*g* for 1 min to pellet hepatocytes. The remaining cells in the supernatant were then harvested and purified using density gradient prepared from Optiprep (Sigma, UK). Mononuclear cells at the interface were collected, washed and erythrocytes in the cell pellet were lysed by incubation with ACK lysis buffer (Lonza, Switzerland). Cells were then blocked with normal mouse serum (Sigma, UK) and were phenotypically characterized by staining with antibodies (Supplementary Table 1).

Fixable Viability Dye eFluor 506 (eBioscience) was used to determine cellular viability. Analysis was performed on an LSR Fortessa flow cytometer (BD Biosciences, UK) using Flowlogic 600.0A software (Inivai Technologies).

4. Flow cytometry of human circulating and hepatic immune cells.

Monocytes, neutrophils and HMCs were incubated with mouse anti-human monoclonal antibodies (Supplementary Table 2) for 20 min at room temperature in the dark and then assessed by flow cytometry for surface marker expression. Cells were washed and re-suspended in phosphate-buffered saline supplemented with 1% fetal bovine serum. Cells were fixed and permeabilized for intracellular cytokine staining (BD Biosciences, UK). TNF-α and IL-6 levels (BD Biosciences, UK) were also determined by flow cytometry using intracellular cytokine staining (BD Biosciences, UK) following 6h stimulation with LPS (100 ng/ml) (Sigma-Aldrich, UK).

Surface Marker	Company	Catalogue Number
F4/80 - AlexaFluor 488	Serotec, UK	MCA497A488T
CD11b - PE-CF594	BD Biosciences, UK	562317
Ly6C - PE-Cy7	eBioscience, UK	25-5932
CD 45.2 - eFluor 450	eBioscience, UK	48-0454
FVD –eFluor 506	eBioscience, UK	65-0866
Ly6G - BV 605	BD Biosciences, UK	563005
MerTK - PE	eBioscience, UK	12-5751
MHC II - eFluor 780	eBioscience, UK	47-5321

Supplementary Table 1. Antibodies used for flow cytometry in murine immune cells. APC, allophycocyanin; FITC, fluorescein isothiocyanate; PE, phycoerythrin.

Surface Marker	Source	Catalogue Number
CD14 - PE-Cy7	BD Biosciences, UK	557742
CD16 - APCH7	BD Biosciences, UK	560195
CD64 - FITC	BD Biosciences, UK	555527
CD86 - APC	BD Biosciences, UK	555660
CD163 - PE	BD Biosciences, UK	556018
CCR2 – AlexaFluor 647	BD Biosciences, UK	558406
CCR5 - FITC	BD Biosciences, UK	555992
CCR7 – FITC	BD Biosciences, UK	561271
HLA-DR - PerCP	eBioscience, UK	9043-9952-120
CD32 - FITC	eBioscience, UK	11-0329-42
CX3CR1 - PE	eBioscience, UK	12-6099-42
MerTK - APC	R&D Systems, UK	FAB8912A
Tie-2 - PE	R&D Systems, UK	FAB3131P

Supplementary Table 2. Antibodies used for flow cytometry in human immune cells. APC, allophycocyanin; FITC, fluorescein isothiocyanate; PE, phycoerythrin.

5. nCounter gene expression analysis

FACS-separated cells from HC and ALF patients (n=3 each) were lysed in RLT lysis buffer (Qiagen, Dusseldorf, Germany) (max 50.000 cells / 2µl) and were stored at -80°C. The cell lysates were incubated with reporter and capture probe sets overnight and afterwards were immobilized on a cartridge using the nCounter® Prep Station (NanoString Technologies, Inc., Seattle, WA). Cartridges were scanned on the nCounter® Digital Analyzer at 600 fields of view at the UCL Nanostring Facility (UCL, London, UK). The differential gene expression was calculated and plotted as heatmap

using the nSolver™ Analysis Software 3.0 (NanoString Technologies, Inc., Seattle, WA). Statistically relevant results are considered with $P < .05$ and a fold-change of 50% higher or lower.

Sample	# MerTK +	# MerTK -	monocyte MerTK (%)
**HC_1**	3.402	318.016	4.0
**HC_2**	4.030	126.477	12.5
**HC_3**	6.071	203.883	10.3
**ALF_1**	12.871	100.449	40.5
**ALF_2**	20.749	342.019	18.0
**ALF_3**	39.233	320.090	34.0

Supplementary Table 3. Data show levels of MerTK monocyte expression and absolute numbers of FACS-separated MerTK+ and MerTK- monocytes from HC (n=3) and ALF patients (n=3).

6. UPLC-MS of acetaminophen and metabolites in mouse plasma.

Mouse plasma samples were analyzed using a modification of a method validated for rat plasma (Dargue et al, manuscript in preparation) as follows. **Chemicals and reagents.** Acetaminophen (paracetamol, APAP) and its glucuronide (APAP-Gluc) conjugate and the deuterated internal standard APAP-d3 were purchased from Sigma Aldrich (Gillingham, UK); its sulphate (APAP-Sulf), cysteinyl (APAP-Cys), N-acetylcysteinyl (APAP-NAC) and glutathione (APAP-GSH) conjugates and the deuterated internal standards, APAP-sulphate-d3, APAP-glucuronide-d3, APAP-cysteinyl-d5 and APAP-NAC-d5, APAP-GSH-d3 were purchased from Toronto research chemicals (Toronto, Canada). Optima grade water was obtained from Fisher Scientific (Leicester, UK), LC-MS grade solvents and formic acid were from Sigma Aldrich (Gillingham, UK).

Sample Preparation. Plasma samples (5µl) were mixed with internal standard stock solution in MeOH (10µl) and MeOH (10µl) to precipitate proteins, were kept at -20°C for 20 min and then centrifuged at 10,000g for 10 min, with 10µL of the clear supernatant mixed with 40 µL water in glass vials for analysis. Samples were quantified using a linear standard curve with weighting 1/x prepared in blank plasma. Ranges of quantification on the instrument were as follows, APAP: 16-500ng/mL, APAP-G and APAP-S: 3.2-100ng/mL, APAP-C and APAP-GSH: 0.64-20ng/mL and APAP-NAC: 0.96-20ng/mL.

UPLC-MS. Chromatographic separation was performed on an Acquity Chromatography system using a 2.1 x 100mm 1.8µm 130 A C18 ACQUITY HSS T3 column (Waters Corporation, Manchester, UK) with a multilinear reversed-phase gradient with mobile phases consisting of water and 0.1% (v/v) FA (solvent A) and methanol and 0.1% (v/v) FA (solvent B). The gradient was performed over a 7.5 min at a flow rate of 0.6mL/min at 40°C. A multi-linear gradient program was employed with the starting conditions set at 5% solvent B for 0.5 min, increasing to 7% at 1.85 minutes then to 8% at 1.9 min, then 10% at 2.5 min, 16% at 4.0 min, 25% at 5 min increasing rapidly to 95% at 5.1 min to wash the column. The solvent composition was held at 95% B for 0.9 minutes before returning to 5% B at 6.1 min. for re-equilibration (1.4 min). The resulting analysis time was 7.5 min/sample. Under these conditions the retention times of acetaminophen and its metabolites were as follows: APAP-Gluc = 1.64 min; APAP-Sulf = 1.97 min; APAP-Cys = 2.38 min; APAP = 2.74 min; APAP-GSH = 3.71 min; APAP-NAC = 4.83 min. In between samples the sample loop was subject to both weak and strong washes of 95:5 water/acetonitrile (v/v) and 100% isopropanol, respectively.

MS/MS data were acquired using a Waters Xevo tandem quadruple (TQ)-S mass spectrometer (Waters Corporation, Manchester, UK). MS/MS Detection was via electrospray ionization (ESI) in positive ion mode using multiple reaction monitoring for the quantification of each compound. Nitrogen was used as the desolvation gas and argon was used as the collision gas. The following generic source conditions were used: capillary voltage, 3 kV; source offset, 30 V; desolvation temperature, 500 °C; source temperature, 150 °C, desolvation gas flow, 1000 L/hr; cone gas flow, 150 L/hr; nebulizer gas, 7.0 bar; collision gas, 0.13 mL/min.

7. Immunohistochemistry, multispectral analysis and confocal microscopy.

Single-epitope enzymatic immunohistochemistry for detection of MERTK, CD163, HLA-DR and MPO expressing cells. FFPE tissue was cut at 4 μm using a Leica RM2235 rotary microtome (Leica Biosystems, UK) and picked up on poly-L-lysine coated slides which were manually stained using a) rabbit monoclonal anti-MERTK primary antibody (# ab52968, Abcam, UK; dilution 1:500), b) mouse monoclonal anti-HLA-DR antibody (# M0746, Dako, UK; dilution 1:100), c) mouse monoclonal anti-CD163 primary antibody (# NCL-L-CD163, Leica Biosystems, UK; dilution 1:100) d) rabbit polyclonal anti-MPO primary antibody (# ab9535, Abcam, UK; dilution 1:50). All slides were dewaxed in xylene, rehydrated, subjected to heat-induced epitope retrieval (HIER) using sodium citrate buffer, pH 6, for 20 min, and allowed to cool, followed by 1 h incubation at room temperature with the primary antibody. The signal was detected using EnVisionTM G/2 Doublestain System, Rabbit/Mouse (DAB+/Permanent Red) (# K536111-2, Dako, UK), and visualized with the Vector VIP peroxidase kit (# SH-600, Vector Laboratories, UK). The slides were then dehydrated with alcohol, cleared with xylene and cover slipped with DPX (Leica Biosystems, UK) after hematoxylin

counterstaining. Images were captured with a Nikon Eclipse E600 microscope using the Nuance™ 3.0.2 (PerkinElmer, UK) multispectral imaging technology.

Double-epitope enzymatic immunohistochemistry for detection of MERTK/CD163 and MERTK/HLA-DR expressing cells. FFPE tissue was cut at 4 μm using a Leica RM2235 rotary microtome (Leica Biosystems, UK) and picked up on poly-L-lysine coated slides which were manually stained using a) rabbit monoclonal anti-MERTK primary antibody (# ab52968, Abcam, UK; dilution 1:300) and mouse monoclonal anti-CD163 primary antibody (# NCL-L-CD163, Leica Biosystems, UK; dilution 1:100), b) rabbit monoclonal anti-MERTK primary antibody (# ab52968, Abcam, UK; dilution 1:300) and mouse monoclonal anti-HLA-DR antibody (# M0746, Dako, UK; dilution 1:100). Slides were dewaxed in xylene, rehydrated, subjected to heat-induced epitope retrieval (HIER) using sodium citrate buffer, pH 6, for 20 min, and allowed to cool, followed by 1 h incubation at room temperature with either the anti-HLA-DR or anti-CD163 antibody. The signal was detected using the EnVisionTM G/2 Doublestain System, Rabbit/Mouse (DAB+/Permanent Red) (# K536111-2, Dako, UK), with Permanent Red for visualization. The slides were then incubated for 1 h at room temperature with the anti-MERTK antibody, and the second signal was detected using the same detection kit but with the Vector VIP peroxidase kit (# SH-600, Vector Laboratories, UK) for visualization. Slides were then dehydrated with alcohol, cleared with xylene and cover slipped with DPX (Leica Biosystems, UK) after hematoxylin counterstaining.

MERTK/CD163 and MERTK/HLA-DR double epitope fluorescent immunostaining. FFPE tissue cut at 4 μm using a Leica RM2235 rotary microtome (Leica Biosystems, UK) was picked up on poly-L-lysine coated slides which were manually stained using a) rabbit monoclonal anti-MERTK primary antibody (# ab52968, Abcam, UK; dilution 1:250)

and a mouse monoclonal anti-CD163 antibody (# NCL-L-CD163, Leica Biosystems, UK; dilution 1:100) or b) rabbit monoclonal anti-MERTK primary antibody (# ab52968, Abcam, UK; dilution 1:250) and a mouse monoclonal anti-HLA-DR antibody (# M0746, Dako, UK; dilution 1:100). Slides were dewaxed in xylene, rehydrated, subjected to heat-induced epitope retrieval (HIER) using sodium citrate buffer, pH 6, for 20 min, and allowed to cool, followed by 15 mins avidin and biotin blocking steps using an avidin/biotin blocking kit (# SP-2001, Vector Laboratories, US), and a 1h incubation with 3% skimmed milk in PBS. Slides were first incubated for 1.5 h at room temperature with the anti-MERTK antibody, followed by an incubation in the same conditions with a biotinylated goat-anti-rabbit secondary antibody (# E043201-8, Dako, UK; dilution 1:200), and another incubation with Alexa Fluor 488-conjugated streptavidin (# S32354, Life Technologies, UK; dilution 1:200). The second primary antibody was then applied (1.5 h at room temperature) followed by an incubation in the same conditions with an Alexa Fluor 594-conjugated goat anti-mouse antibody (# A11032, Life Technologies, UK; dilution 1:200). All slides were counterstained with 4′,6-diamidino-2-phenylindole (DAPI) (# D1306, Life Technologies, UK), cover slipped using a fluorescence mounting medium (# S302380-2, Life Technologies, UK) and imaged using a Zeiss LSM800 confocal microscope (Zeiss, UK).

TUNEL/MPO double-epitope fluorescent immunostaining. FFPE tissue cut at 4 μm using a Leica RM2235 rotary microtome (Leica Biosystems, UK) was picked up on poly-L-lysine coated slides which were manually stained using an apoptosis staining kit (# C10618, Molecular Probes, UK), according to manufacturer's instructions, followed by an immunofluorescent stain using a rabbit polyclonal anti-MPO antibody (# ab9535, Abcam, UK; dilution 1:25). After TUNEL staining, the slides were incubated overnight at 4°C with the anti-MPO antibody. The second day the slides were incubated for 2 hours

at room temperature with a biotinylated goat-anti-rabbit secondary antibody (# E043201-8, Dako, UK; dilution 1:100). This was followed by a 1hour incubation at room temperature with Alexa Fluor 488-conjugated streptavidin (Life Technologies, UK; dilution 1:100). All slides were counterstained with 4′,6-diamidino-2-phenylindole (DAPI) (# D1306, Life Technologies, UK), cover slipped using a fluorescence mounting medium (# S302380-2, Life Technologies, UK) and imaged using a Zeiss LSM800 confocal microscope (Zeiss, UK).

Assessing MERTK/HLA-DR and MERTK/CD163 expression - Nuance multispectral imaging and confocal microscopy. Double-epitope enzymatic immunohistochemistry (MERTK/CD163, MERTK/HLA-DR, MPO/TUNEL) was used to assess co-localization. Distinguishing between red and purple and unmixing these co-localized chromagens is not feasible with an RGB camera as it only uses 3 channels, so a multispectral approach was used. Nuance camera takes several images over different wavelengths, thus identifying the ‘individual spectral signature’ of each chromagen, saving and unmixing it from adjacent colours. Once unmixed, images are re-coloured, enabling distinction of chromogens and inverting to a pseudo-fluorescence image. In addition, confocal examination of double epitope fluorescent immunostains was performed to confirm MERTK/HLA-DR, MERTK/CD163 and MPO/TUNEL cells co-expression, to overcome a few obstacles such as the thickness of the histological sections which allowed for the overlapping of signals.

8. Cell culture experiments.

Hepatic mononuclear cells and CD14-isolated monocytes were cultured for 48h at 10^6 cells/ml in 24-well plates (Corning, USA) (37°C in 5% CO_2) in fresh complete medium [RPMI-1640 medium containing 10% heat-inactivated FBS medium and 1% antibiotics (L-glutamine, penicillin, and streptomycin)] (all from Life Technologies, UK). Neutrophils were cultured for 6h or 20h at 10^6 cells/ml as in the above conditions. Effects of recombinant human (rh)-SLPI (R&D Systems, UK) (0 and 0.5 μg/mL) in these cell culture experiments were assessed. All cells were harvested and washed in sterile phosphate-buffered saline before any further analysis.

9. Measurement of cytokines.

Human quantikine ELISA was used to measure SLPI and cytokine levels in cell culture supernatants (R&D Systems, UK). ELISA microplates were read using the SoftMax® Pro software (Molecular Devices, USA). Meso Scale Discovery (MSD) human pro-inflammatory 10-plex panel kit was used in BEC supernatants. MSD plates were read on the Sector Imager 2400 (Gaithersburg, MD).

10. Monocyte migration assay.

Collagen plugs containing recombinant human (rh)-SLPI (0 and 0.5 μg/mL) (R&D Systems, UK) were formed in cell culture inserts (Merck Millipore, Germany) placed in 24-well plates (Corning, USA). Human hepatic sinusoidal endothelial cells (HSECs) (5×10^4) were added/grown on the plugs and then were stimulated with TNF-α and IFN-γ (both 10 ng/ml) (R&D Systems, UK) (37°C in 5% CO_2). CD14-isolated healthy monocytes ($2x10^6$) were added on top of the collagen matrix and after 1.5h incubation non-migrated monocytes were harvested. Sub-endothelial monocytes were recovered

from the HSEC/collagen matrix after 24h, following incubation with collagenase solution, as previously (27). Monocyte phenotype was determined and cells (cells/µl) were quantified using Count Bright Absolute Counting Beads by flow cytometry (Life Technologies, UK).

***11.* Apoptosis, phagocytosis and oxidative burst.**

Apoptosis. Cell apoptosis kit (BD Biosciences, UK) was used to quantitatively determine the percentage of neutrophils in culture actively undergoing apoptosis. Discrimination of total apoptotic cells (Annexin-V+), early apoptotic cells (7-AAD-Annexin-V+) and end stage apoptotic cells (7-AAD+Annexin-V+) was based on 7-AAD and Annexin-V staining. Phagocytosis and oxidative burst. Neutrophil oxidative burst was assessed using the Phagoburst kit (Glycotrope, Germany) and monocyte phagocytosis using the pHRodo kit (ThermoFisher Scientific, UK), both by flow cytometry and according to the manufacturer's instructions.

***12.* Neutrophil extracellular traps (NETs).**

NET formation and extracellular DNA quantification. Neutrophils (2 x 10^5) were stimulated with 25 nM PMA (Sigma-Aldrich, UK) or 100 ng/mL LPS (Sigma-Adlrich, UK) in the absence and presence of (rh)-SLPI (0, and 0.5 µg/mL) (R&D Systems, UK) for 3 hours, 37°C, 5% CO_2. Supernatants were then removed and centrifuged at 2,200x*g* for 10 min. Extracellular DNA content was analyzed by incubation with 5µM SYTOX Green Dye (Life Technologies, UK) for 10 min and fluorometrically examined using a BioTek Synergy HT plate reader (NorthStar Scientific Ltd, UK) at 485/528nm. Samples were calibrated to a λ-DNA (Fisher Scientific, UK) standard curve. *NET visualization by fluorescent microscopy*. Neutrophils (2 x 10^5) were seeded onto glass coverslips (VWR International, UK) and were stimulated with 25 nM PMA (Sigma-Adlrich, UK) or 100

ng/mL LPS (Sigma-Aldrich, UK) with and without the presence of (rh)-SLPI (0, and 0.5 μg/mL) (R&D Systems, UK) for 3 hours, 37°C, 5% CO_2 before fixation with 4% paraformaldehyde. Neutrophils were then permeabilised with 0.1% Triton X-100, stained with 1μM SYTOX Green Dye (Life Technologies, UK), fixed with fluoromount medium and visualized by Zeiss Axiovert 200M microscope (Carl Zeiss Ltd, UK).

13. Effects of blocking SLPI in cell culture supernatants.

CD14-isolated monocytes were cultured for 48h at 10^6 cells/ml in 24-well plates (Corning, USA) (37°C in 5% CO_2) in fresh complete medium or 25% human plasma samples from ALF (n=10) patients, which were pre-incubated with anti-human SLPI neutralizing antibody (R&D Systems, UK) (0 and 5 μg/ml) for 1h at room temperature, before addition to the healthy monocyte cultures. The anti-human SLPI blocking antibody we used was a referenced neutralizing antibody from the manufacturer, as previously (7). Effects of the ALF plasma-conditioned monocyte culture supernatants on healthy neutrophil survival and function were examined

14. Induction of apoptosis and monocyte efferocytosis of apoptotic cells.

Human neutrophils were isolated as described above and were re-suspended at 10^6 cells/ml in medium, labeled with CellTracker Green CMFDA (Life Technologies, UK) (5 μM in serum-free medium, 45 min, dark) and incubated for 20h (37℃ in 5% CO_2) in 24-well plates (Corning, USA). Annexin V kit (BD Biosciences, UK) was used to quantitatively determine the percentage of neutrophils in culture that are actively undergoing apoptosis. The composition of neutrophils obtained after incubation was over 70% apoptotic cells, according to Annexin V staining (BD Biosciences, UK).

Huh-7 hepatoma cells were cultured in DMEM medium (containing 1% PenStrep, 1%

NEAA, pyruvate, 1% L-glutamine and 10% FBS) (Life Technologies, UK) in vented culture flasks under normal culture conditions (37°C, 5% $C0_2$). Cells were passaged no more than 3 times before used in experiments. Huh-7 cells were seeded on glass cover slips in 24-well plates and allowed to grow until confluent (~$0.2x10^6$). Huh-7 cells were washed once with PBS and then were labeled with CellTracker Green CMFDA (Life Technologies, UK) (5 µM in serum-free DMEM, 45 min, dark). Cells were washed again and then were given 300 µl serum-free DMEM containing 20 µM staurosporine (STS) (Sigma-Aldrich, UK) or an equal volume of DMSO. Cells were cultured for 16h and then were fixed with 1% PFA (Sigma-Aldrich, UK) in PBS for 5 min, and mounted on microscope slides with Prolong Gold antifade mounting reagent (Life Technologies, UK). Cells were imaged using a Zeiss LSM 780 microscope at x40 magnification using 0.6 zoom or x63 magnification under normal zoom.

Human primary hepatocytes (Life Technologies, UK) were first thawed using Cryopreserved Hepatocyte Recovery Medium (CHRM) (Life Technologies, UK) in order to determine cell count and viability using trypan blue staining. Cells were then plated using CHPM medium in a 96-well collagen pre-coated plates at a $0.9x10^6$ cells/ml density for 4 h to allow cell adherence, before wash and replacement of CHPM with Williams' E supplemented medium (Life Technologies, UK). Hepatocytes were allowed to further adhere overnight. Cultures were then checked for their cell morphology and monolayer integrity before being washed and then be supplemented with fresh medium only or medium containing 20 mM acetaminophen (APAP) (Sigma-Aldrich, UK). After completion of the 24h treatment period, cells were washed and pre-labeled with CellTracker Green CMFDA (Life Technologies, UK) (5 µM in serum-free medium, 45 min, dark) before co-culture with monocytes.

Supplementary Figures

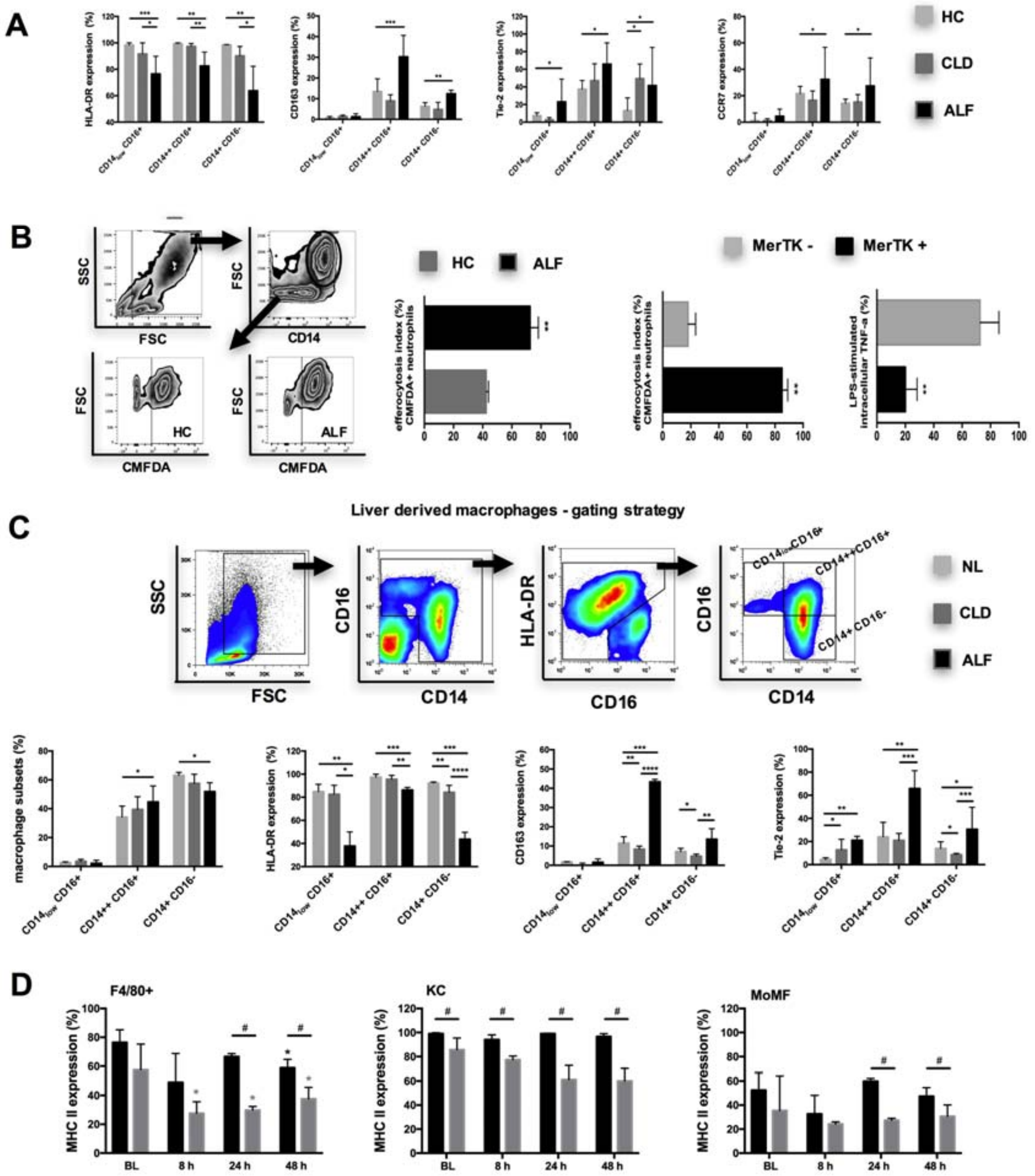

Suppl. Fig. 1. Characterization of monocytes and liver-derived macrophages in human ALF. (A) Data show the percentage of expression of activation (HLA-DR), pro-resolution (CD163), pro-angiogenesis (Tie-2) and lymph-node homing (CCR7) markers in monocyte subsets (classical, intermediate and non-classical) in ALF (n=15), CLD (n=10) patients and HC (n=15). **(B)** (left) HC and ALF PBMCs were co-cultured (4h) with CMFDA-labeled apoptotic neutrophils. Representative flow cytometry analysis of CMFDA-labeled neutrophils phagocytosed by CD14+ monocytes are shown (n=6 each). (right) Proportion of MerTK+ vs MerTK- monocytes that

phagocytosed CMFDA-labeled neutrophils and that were stained positive for intracellular TNF-α (LPS 100ng/ml). **(C)** (upper) Representative flow cytometry analysis and gating strategy used to identify total liver derived macrophages and their subsets, isolated from ALF (n=8), CLD (n=10) and normal (NL, n=6) liver. (lower) Percentage of macrophage surface marker expression in different groups. **(D)** WT (black bars) and $Mer^{-/-}$ (grey bars) mice dosed with APAP were studied at 8, 24 and 48 hours and untreated mice served as baseline (n=4/group). Data show the MHC class II expression of F4/80+ hepatic macrophages, Kupffer cells (KC) and monocyte-derived macrophages (MoMF). Non-parametric (Mann-Whitney) statistical analysis was used. Data are expressed as median values with interquartile range (IQR). *or # P< .05, ** P< 0.01, *** P< .001, **** P< .0001.

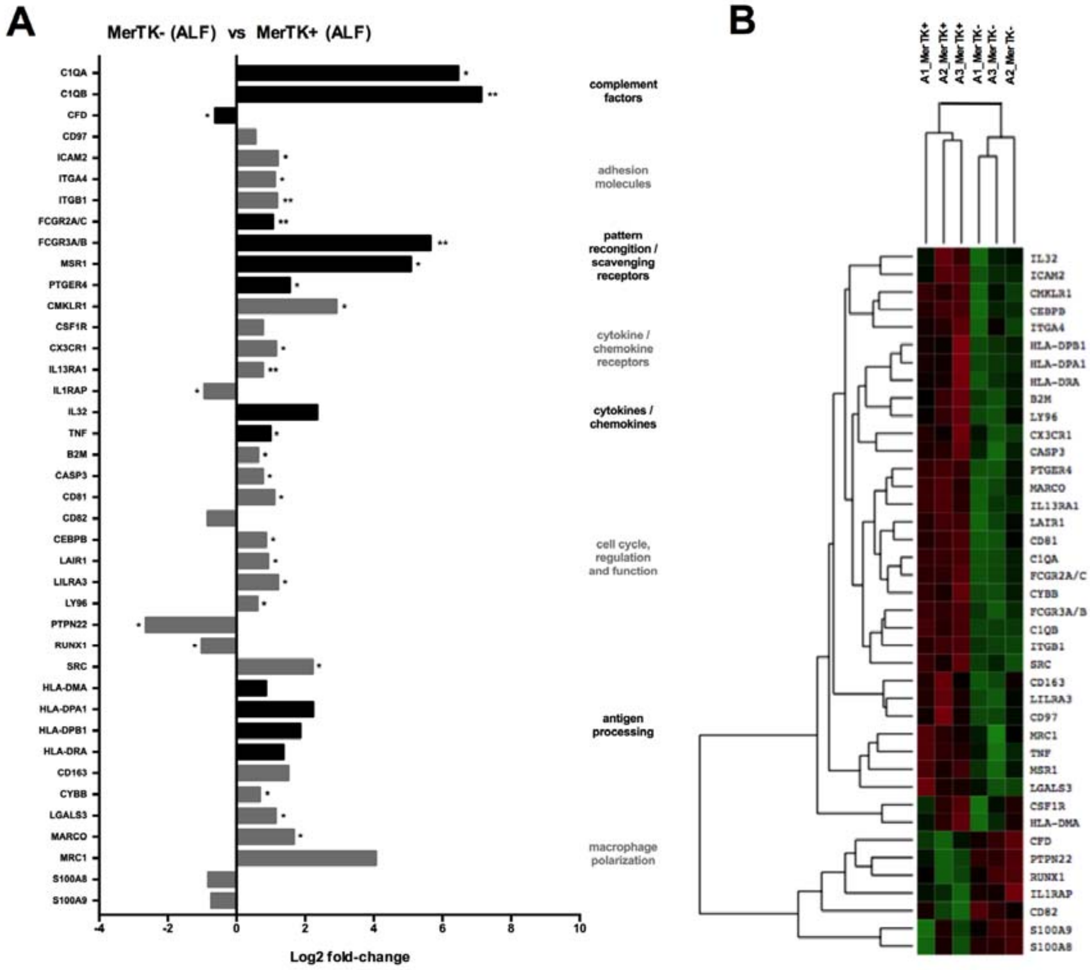

Suppl. Fig. 2. Gene expression pattern of MerTK+ vs MerTK- monocytes in ALF patients. MerTK+ and MerTK- monocytes were FACS-sorted from ALF PBMCs (n=3). Highly pure isolates of the MerTK± subsets were subjected to quantitative microarray gene expression analysis (nCounter® GX Human Immunology V2 kit, profiling 594 immunology-related human genes; NanoString Technologies, Inc., Seattle, WA). **(A-B)** Data show the Log2 fold-change and agglomerative cluster (heatmap, z-Score; green=min and red=max magnitude of expression) of 35 differentially expressed genes, comparing MerTK+ vs MerTK- monocytes in ALF patients. * p< .05, ** p< 0.01, *** p< .001.

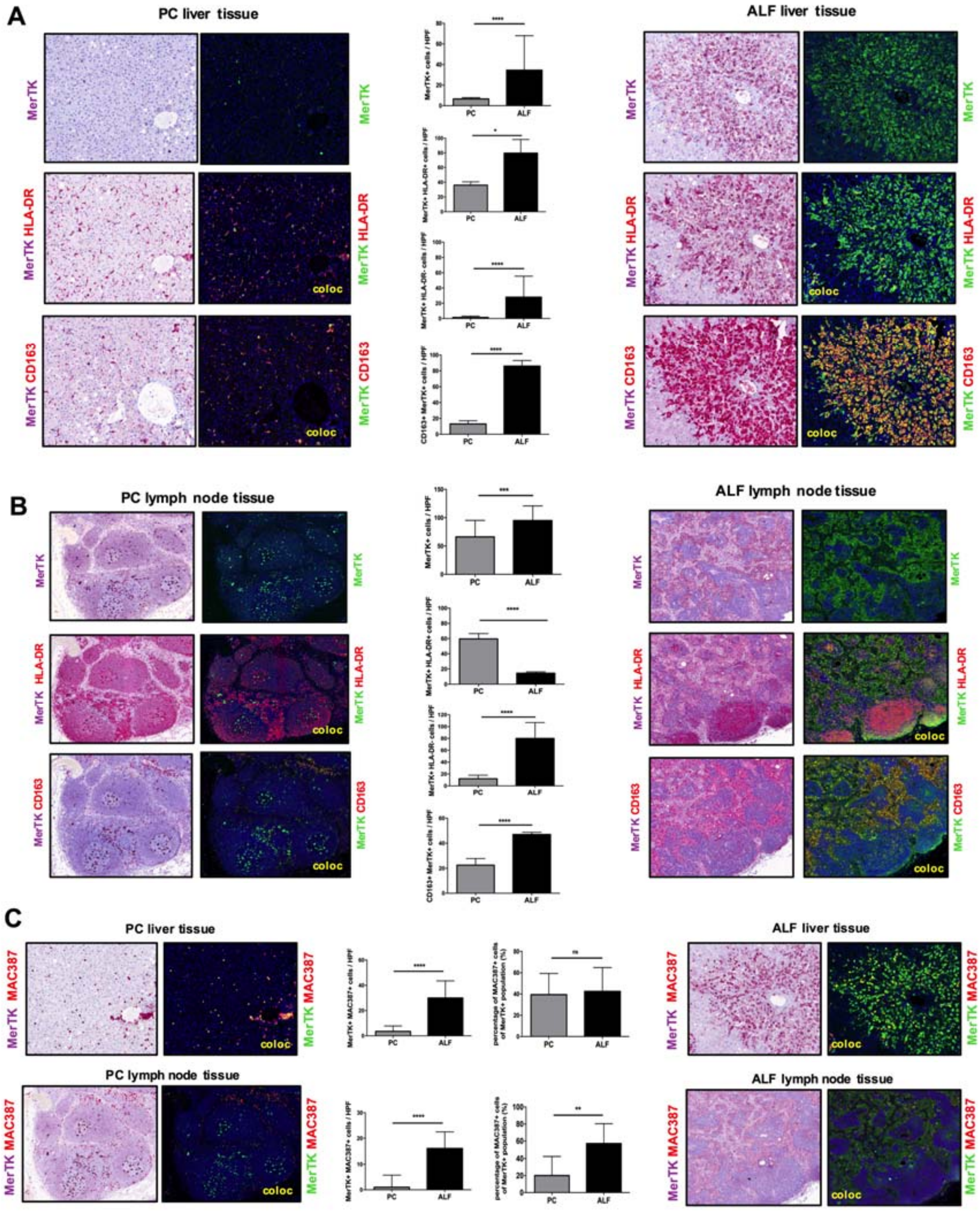

Suppl. Fig. 3. Resolution-like MerTK+ macrophages are expanded in the liver and mesenteric lymph nodes of ALF patients. (A) Representative immunohistochemistry (IHC) images and enumeration of MerTK+, MerTK+HLA-DR± and CD163+MerTK+ cells [positive cells per 5 high-power field (HPF)] in centrilobular areas of pathological control (PC, n=4) and acute liver failure (ALF, n=6) liver tissue using IHC and multispectral image analysis. RGB images

(200X) show MerTK+ (purple), HLA-DR+/CD163+ (red) cells and pseudofluorescent images show MerTK+ (green), HLA-DR+ (red), CD163+ (red) and co-localization (yellow). **(B)** Representative IHC images and enumeration of MerTK+, MerTK+HLA-DR±, and CD163+MerTK+ + cells [positive cells per 5 high-power field (HPF)] in mesenteric lymph node tissue from PC (left, n=4) and ALF (right, n=4) patients. For both groups: (left panel) RGB images (100X) showing MerTK+ (purple) and HLA-DR+/CD163+ (red) cells. (right panel) pseudofluorescent images (100X) show MerTK+ (green), HLA-DR+/CD163+ (red) cells and their co-localization (yellow). **(C)** Representative IHC images and data showing enumeration of MAC387+MerTK+ cells and proportion of MAC387+ within the total MerTK+ population in PC/ALF liver and lymph node tissue, using IHC and multispectral image analysis. RGB images (200X) show MerTK+ (purple) and MAC387+ (red) cells and pseudofluorescent images show MerTK+ (green), MAC387+ (red) and co-localization (yellow). Non-parametric (Mann-Whitney) statistical analysis was used. Data are expressed as median values with interquartile range (IQR). * $p < .05$, ** $p < 0.01$, *** $p < .001$, **** $p < .0001$

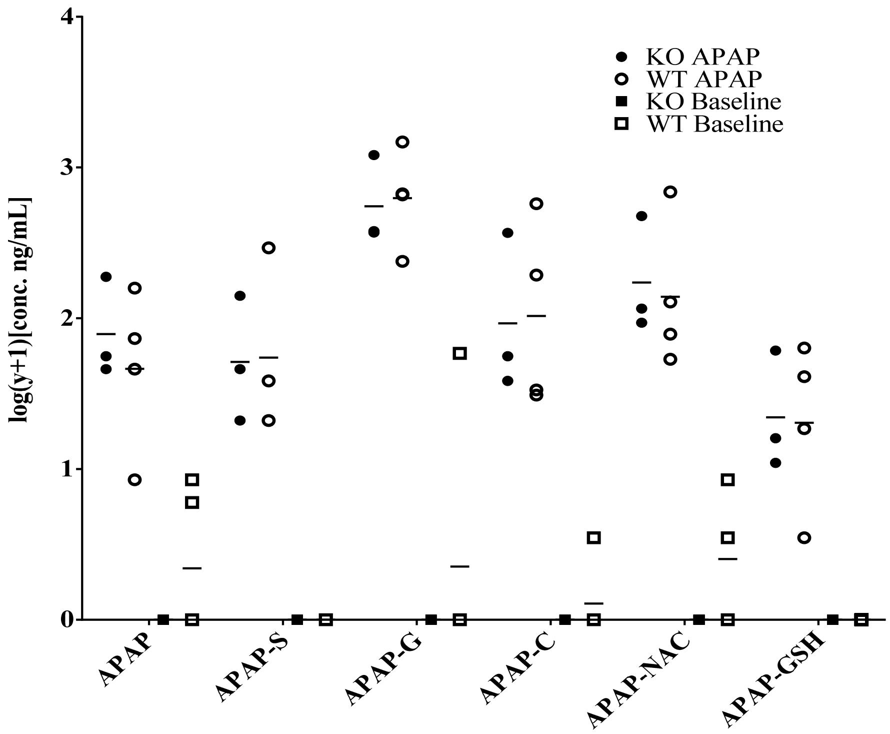

Suppl. Fig. 4. UPLC-MS of acetaminophen and metabolites in plasma samples of WT and Mer$^{-/-}$ mice. Wild-type (WT) and Mer-deficient mice (KO) mice dosed with acetaminophen (APAP, 300mg/kg) were studied at 8 hours, while untreated mice served as baseline controls. Mouse plasma samples derived from both study groups were analysed for APAP and five metabolites [APAP-glucuronide (APAP-G), APAP-sulfate (APAP-S), APAP-cysteinyl (APAP-C), APAP-glutathione (APAP-GSH) and APAP-N-acetylcysteinyl (APAP-NAC)], Data show the detected log transformed [y=log(y)+1)] concentrations (ng/mL) for APAP and each metabolite [KO APAP (n=3), WT APAP (n=4), KO baseline (n=4), WT baseline (n=5)]. Non-parametric analyses revealed that concentrations (two tailed, Mann-Whitney test, p=0.6753) or log transformed concentrations (two tailed t-test, p=0.8820) were not significantly different between WT and KO animals.

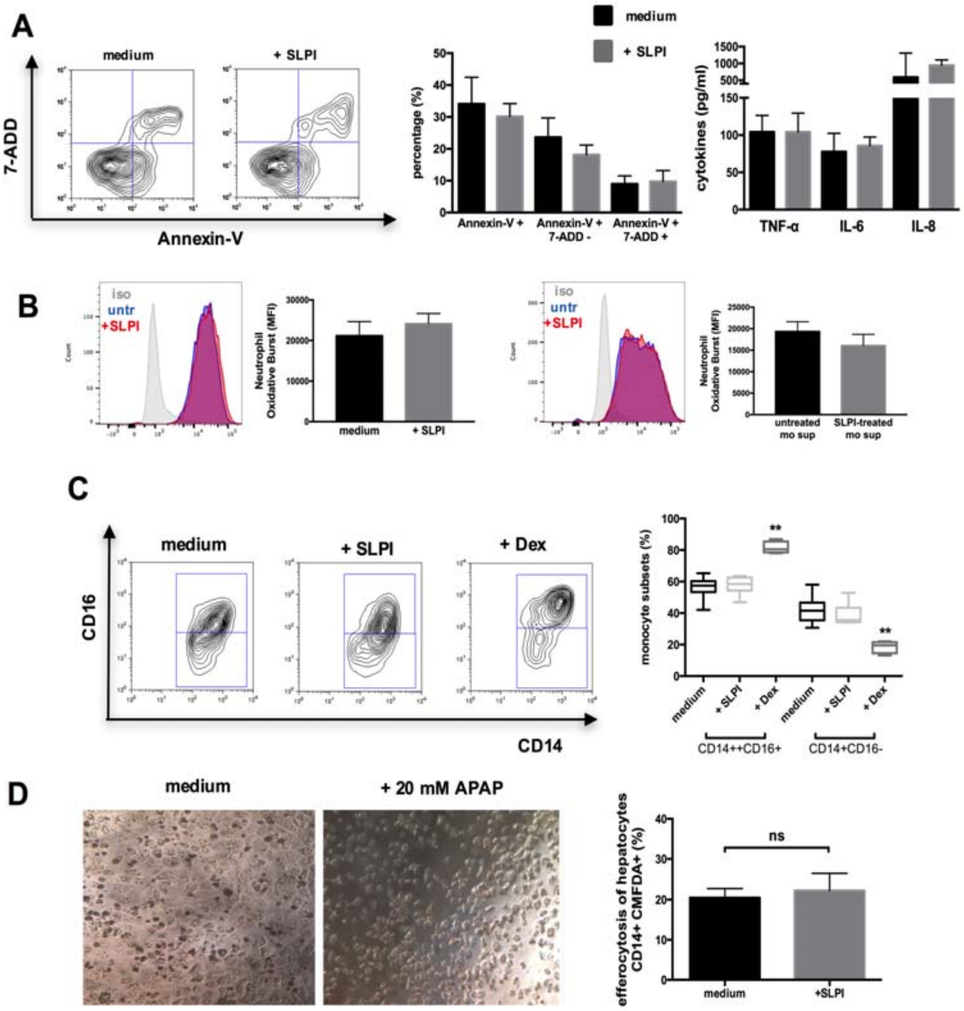

Suppl. Fig. 5. Effects of SLPI on neutrophils and monocyte efferocytosis. (A-B) Effects of (rh)-SLPI (0 and 0.5 μg/ml) and paracrine effects of ±SLPI-treated monocyte supernatants on neutrophils were examined (n=5). **(A)** (left) Representative Annexin-V/7-AAD staining and percentage of apoptotic neutrophils after 6h culture period; (right) LPS-stimulated (LPS 100ng/ml) cytokine levels (pg/ml) in culture supernatants, as measured by ELISA. **(B)** Representative histograms of neutrophil oxidative burst in response to *E. coli* after treatments (3h). Results expressed as mean fluorescence intensity (MFI). **(C)** Representative flow cytometry and data showing the percentage of CD14+CD16- and CD14++CD16+ monocytes after 48h culture with different treatments [medium, SLPI (0.5 μg/ml), Dexamethasone (100 nM)]. Results shown as percentage for positive cells. **(D)** (Left) Data show the percentage of CD14-labeled monocytes treated with/without SLPI that phagocytosed CMFDA-labeled APAP-treated (20mM) apoptotic primary hepatocytes (n=2). (Right) Light microscopy images of hepatocytes cultured with/without acetaminophen (APAP, 20mM). Non-parametric (Mann-Whitney) statistical analysis was used. Data are expressed as median values with interquartile range (IQR). * P< .05, ** P< 0.01.

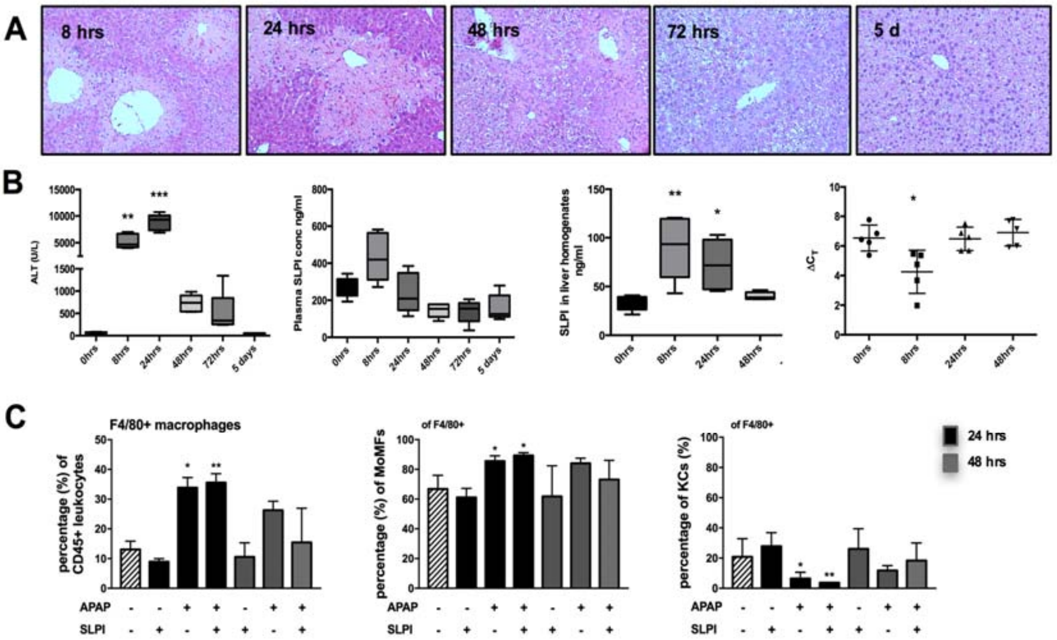

Suppl. Fig. 6. SLPI and APAP-induced liver injury in mice. (A-B) WT mice treated with APAP were studied at several time points (up to 5 days) while untreated mice served as control group. **(A)** Representative images of liver sections stained with H&E describing the evolution of liver damage in APAP-mice. **(B)** Plasma ALT levels (U/L) (Kruskal-Wallis test used), plasma SLPI levels (ng/ml) (Dunn's test used), liver SLPI protein (ng/ml) (Dunn's test used) and liver SLPI mRNA (ΔCt) levels as determined by RT-PCR (Kruskal-Wallis test used), compared to baselin. **(C)** WT mice were dosed with APAP or SLPI or APAP plus SLPI while untreated mice served as baseline controls (n=6/group). Mice were studied at baseline (white bars), 24 hours (black bars) and 48 hours (grey bars). Data show the F4/80+ hepatic macrophages as proportion (%) of total CD45+ leukocytes and the percentage (%) of F4/80^{high}CD11b_{low} resident Kupffer cells (KC) and F4/80_{low}CD11b^{high} monocyte-derived macrophages (MoMF) within the F4/80+ population (n=6 mice/group). Non-parametric (Mann-Whitney) statistical analysis was used. Data are expressed as median values with interquartile range (IQR). * P< .05, ** P< 0.01, *** P< .001.
